# Electrophilic MiniFrags Revealed Unprecedented Binding Sites for Covalent HDAC8 Inhibitors

**DOI:** 10.1021/acs.jmedchem.3c01779

**Published:** 2023-12-19

**Authors:** Aaron B. Keeley, Aleksandra Kopranovic, Vincenzo Di Lorenzo, Péter Ábrányi-Balogh, Niklas Jänsch, Linh N. Lai, László Petri, Zoltán Orgován, Daniel Pölöske, Anna Orlova, András György Németh, Charlotte Desczyk, Tímea Imre, Dávid Bajusz, Richard Moriggl, Franz-Josef Meyer-Almes, György M. Keserü

**Affiliations:** a Medicinal Chemistry Research Group, Research Centre for Natural Sciences, Magyar tudósok krt 2, H-1117 Budapest, Hungary; b Department of Organic Chemistry and Technology, Faculty of Chemical Technology and Biotechnology, Budapest University of Technology and Economics, Müegyetem rkp. 3., H-1111 Budapest, Hungary; c National Laboratory for Drug Research and Development, H-1117 Budapest, Hungary; d Department of Chemical Engineering and Biotechnology, University of Applied Sciences Darmstadt, Haardtring 100, 64295 Darmstadt, Germany; e Institute of Animal Breeding and Genetics, University of Veterinary Medicine, 1210 Vienna, Austria; f MS Metabolomics Research Group, Research Centre for Natural Sciences, Magyar tudósok krt 2, H-1117 Budapest, Hungary

## Abstract

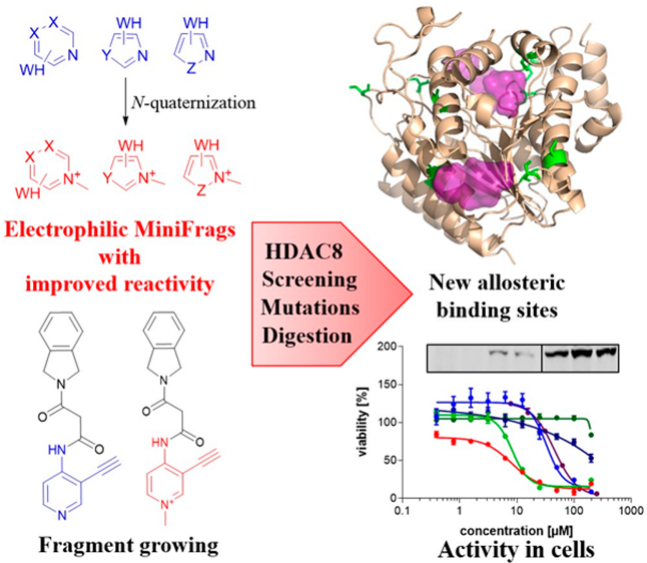

Screening of ultra-low-molecular weight ligands (MiniFrags) successfully identified viable chemical starting points for a variety of drug targets. Here we report the electrophilic analogues of MiniFrags that allow the mapping of potential binding sites for covalent inhibitors by biochemical screening and mass spectrometry. Small electrophilic heterocycles and their N-quaternized analogues were first characterized in the glutathione assay to analyze their electrophilic reactivity. Next, the library was used for systematic mapping of potential covalent binding sites available in human histone deacetylase 8 (HDAC8). The covalent labeling of HDAC8 cysteines has been proven by tandem mass spectrometry measurements, and the observations were explained by mutating HDAC8 cysteines. As a result, screening of electrophilic MiniFrags identified three potential binding sites suitable for the development of allosteric covalent HDAC8 inhibitors. One of the hit fragments was merged with a known HDAC8 inhibitor fragment using different linkers, and the linker length was optimized to result in a lead-like covalent inhibitor.

## Introduction

Crystallographic screening of ultra-low-molecular weight ligands (MiniFrags) was first reported by Astex and defined as an effective tool for the detection of unprecedented ligand binding pockets.^1^ Because the chemical space of the MiniFrags is limited, a small library might provide acceptable coverage and enhanced sampling relative to conventional fragment libraries. The approach was used to identify potential ligand binding sites and hot and warm spots to drive the design strategy of drug discovery programs. To achieve this goal, however, MiniFrags should have been screened at high concentrations with resource intensive X-ray crystallography, and the weak affinity of the fragments made their detection challenging. Our motivation was therefore designing the electrophilic alternative of MiniFrags that (i) could be first screened in biochemical assays, (ii) provide hits with higher potency and more stable binding mode due to covalent labeling, (iii) identify binding sites by readily available mass spectrometry, and (iv) serve as viable starting points for covalent lead-like compounds. These compounds were designed to be heterocyclic fragments with six to nine heavy atoms containing an electrophilic warhead.

Heterocycles are considered as main building blocks of drugs and drug-like compounds due to their ability to interact with the targeted protein.^2−4^ In addition, the emerging field of targeted covalent inhibitors (TCIs) has shown their potential for carrying electrophilic warheads and tuning their reactivity.^5−11^ In particular, we showed that the electron-withdrawing character of the heterocycles can enhance the reactivity of electrophilic functional groups, and therefore, they can be considered as covalent warheads for targeting nucleophilic amino acid residues, mostly cysteine.^12−15^ This reactivity can be further improved by the quaternization of the aromatic nitrogen atom of the aromatic ring, introducing a positive charge that enhances the electrophilic reactivity of the warhead by electron withdrawal. As single examples, it has been shown that the N-methylation of 4-bromo- or 2-vinylpyridine resulted in improved thiol reactivity, and the quaternized heterocyclic electrophile could be used for protein labeling.^13,16^ A similar activating effect was observed for 4,4′-dipyridylsulfides caused by enzymatic protonation of a pyridine nitrogen.^17^ Moreover, comparing a library of nonmethylated and methylated electrophiles against the antibacterial target MurA also showed an increase in potency.^18^ These results together suggest that a designed library of quaternized electrophilic heterocycles might provide relevant information about tractable covalent binding sites, together with suitable starting points for covalent drug discovery programs.

The most popular design strategy of covalent inhibitors involves the attachment of an electrophilic warhead to the appropriate position of a noncovalent binder (ligand-first approach).^19^ The warheads can consist of plenty of functional groups, mainly built from three (e.g., isothiocyanate), seven (e.g., acrylamide), or even more atoms or rings (e.g., maleimide).^6,20,21^ The modification of the noncovalent core with these functional groups evidently changes the original pose at the binding site and influences the binding affinity that urges an iterative optimization strategy. On the contrary, small warheads with only one or two atoms (e.g., a halogen or nitrile, vinyl, or acetylene groups, respectively) can minimize changes in the binding mode. Unfortunately, however, these small functional groups are usually not reactive enough, acting in aromatic nucleophilic substitutions or nucleophilic additions, but via attachment to an electron-withdrawing heterocyclic core, their reactivity can be enhanced. Thus, the formed heterocyclic electrophiles can label protein nucleophiles, e.g., cysteines, successfully.^6,8,11,12,14,15,22,23^ In addition to characterizing potential binding sites, covalently bound heterocycles can be considered as starting points for covalent fragment-based approaches.^13,24^ Realizing these advantages, we aimed to develop and characterize a screening library of covalent MiniFrags as a novel electrophile-first approach against suitable protein targets.

The proposed strategy was first tested on mapping the potential binding sites of human histone deacetylase 8 (HDAC8). HDAC8 is a member of the HDAC enzyme family having an important role in cell cycle progression by catalyzing the deacetylation of histones and a number of cytosolic proteins.^25^ HDACs participate in critical signaling networks, and their deregulation has been linked to many diseases, including cancer by effecting cell reproduction, neurodegenerative disorders, metabolic dysregulation, and autoimmune and inflammatory diseases.^26−29^ HDAC8 has 10 cysteines, and eight of them can in general form four disulfide bridges. Recent studies have shown that Cys102 and Cys153 are redox-sensitive and form an enzyme activity regulating disulfide bridge near the active site, therefore acting as a redox switch.^30^ The other three disulfide bridges (Cys125-S-S-Cys131, Cys244-S-S-Cys287, and Cys275-S-S-Cys352) can be induced after treatment with a sulfenamide-containing inhibitor. It was shown that the disulfide bridge between Cys275 and Cys352 regulates the enzyme activity to ∼50%.^31^ Cys244 is present in only HDAC8 and might be located at a position suitable for allosteric modulation such as Cys28 and Cys314 that are not forming disulfide bonds. Moreover, Cys28 is positioned in helix 1, and it was shown that the helix 1–helix 2 region functions as an allosteric regulative domain; structural perturbations at this region alter the enzyme activity.^32^ Additionally, Cys28 is also unique for HDAC8 by means of structural alignments. Altogether, these studies suggest an inimitable regulative cysteine pattern for HDAC8, which appears to be particularly suitable for testing electrophilic MiniFrags.

In this work, we report the development of an electrophilic heterocyclic fragment library, the covalent MiniFrags. The library was compiled from five- or six-membered heterocycles equipped with six warheads (Cl, Br, I, nitrile, vinyl, and acetylene), and the commercially available subset was subjected to quaternization. We show the effect of the methylation on the aromatic nitrogen atoms enhancing cysteine reactivity and potency by systematically characterizing the library in an HPLC/MS-based thiol reactivity assay and in the HDAC8 biochemical assay. This approach led us to new low-micromolar and also nanomolar HDAC8 inhibitor fragments that could be considered as viable starting points for novel HDAC8 inhibitor chemotypes. Merging one of the MiniFrags with a known HDAC8 inhibitor fragment transformed a reversible inhibitor to an irreversible one, and the significance of the linker length was confirmed. In addition, mutational analysis coupled with MS/MS studies revealed a new set of allosteric sites that are available for covalent targeting. These results and the availability of the covalent MiniFrag library^33^ reported here could initiate further studies in this direction.

## Results

### Quaternized Heterocyclic Electrophiles Show Enhanced Thiol Reactivity

**Scheme 1:**
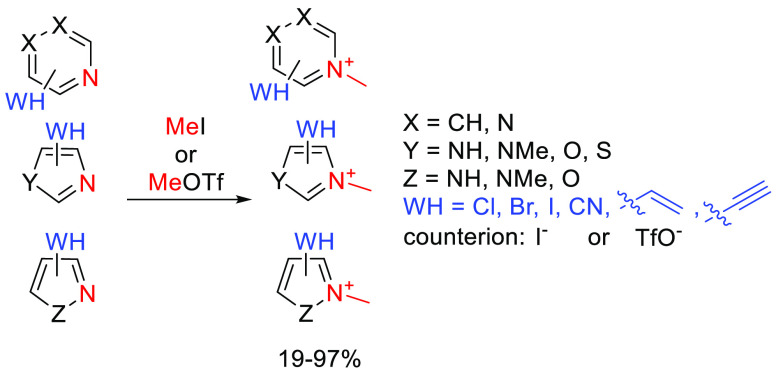
General Scheme for the Methylation of the Heterocyclic Electrophiles The methylated nitrogen atom is colored red, and the warhead is colored blue.

The heterocyclic cores of the covalent MiniFrags were pyridines, pyrimidines, pyrazines, imidazoles, pyrazoles, oxazoles, thiazoles, and isoxazoles substituted at various positions. The electrophilic moieties were the Cl, Br, and I atoms reacting in aromatic nucleophilic substitution, or nitrile, vinyl, and ethynyl groups reacting in nucleophilic addition.^14,15^ Methylation of the aromatic nitrogen was realized using methyl iodide or methyl trifluoromethanesulfonate (Scheme 1). In most cases, the reactions went smoothly, resulting in acceptable yields after a simple filtration or evaporation of the solvent. In the case of imidazoles and pyrazoles, both nitrogen atoms were methylated. For pyrazines, the methylated products were obtained in an equal quantity. The products were iodide or triflate salts, and finally, the library contained 58 compounds in total. We have computed the LUMO energies of the heterocycles and found that in all cases the methylated fragments had lower LUMO energy values (−0.065 ± 0.027 hartree on average) than the nonmethylated ones (−0.019 ± 0.019 hartree on average). The lower orbital levels are closer to the HOMO value of MeS^–^ (−0.227 hartree), supporting the increased reactivity against thiolates. No clear correlation between LUMO energies and experimental reactivities was observed considering the whole library, but among the heterocyclic cores, imidazoles and pyrazoles had the highest LUMO values that were in line with their limited reactivity.

Electrophilic heterocycles were tested in a GSH-based reactivity assay by HPLC-MS. Compounds with a wide range of reactivity were identified, and some reactivity trends could be seen. The reactivity (*t*_1/2_) range 0.1–1377 h, and a compound was considered as reactive if *t*_1/2_ is <48 h. For the comparability of these results with that of the nonmethylated heterocyclic electrophiles,^14,15^ the IDs remained the same in similar figures and in the text the methylated compounds are labeled with a plus.

**Figure 1:**
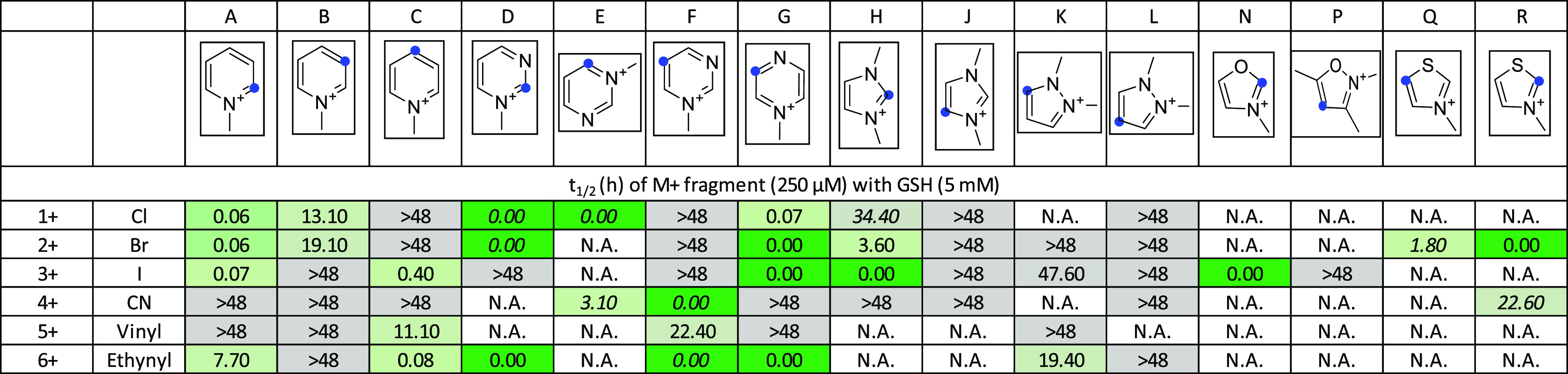
Quaternized heterocyclic electrophiles show enhanced thiol reactivity in the GSH reactivity assay. Blue dots indicate the position of the warhead. The coloring is in line with the activity, as the darker green color refers to a higher reaction rate and a lower half-life. Compounds labeled by italics showed parallel reaction with the assay buffer. N.A. stands for “not available”.

Analyzing the impact of quaternization in depth, we compared the results of the GSH assay of both sets. It turned out that from the nonmethylated set 16 of 84 (19%) were reactive, while from the methylated set, 30 of 58 (52%) were reactive under these conditions. The quaternized compounds reacted in <10 min in 17 cases. Four compounds reacted in <4 h. Five compounds reacted in <20 h. Four compounds reacted in <48 h. Twenty-eight did not react (Figure 1). In comparison, for the nonmethylated pairs, these numbers were 0, 8, 3, 5, and 68, respectively (Figure S1). In the case of pyridiniums, warheads at positions 2 and 4 (Figure 1, columns A and C) showed high reactivity, while compounds with warheads at position 3 (Figure 1, column B) were inactive except for 2-chloro- and 2-bromopyridinium (**B1+** and **B2+**, respectively), which, in general, corresponds with the results obtained for the nonmethylated heterocyclic library. The less active pyridiniums were equipped with the CN warhead (**A4+**, **B4+**, and **C4+**), and in general, the vinyl (Figure 1, row 6) also showed weaker reactivity. Among six-membered heterocycles with two nitrogen atoms, 2- and 4-pyrimidiniums (Figure 1, columns D and E) and two pyraziniums (Figure 1, column G) reacted rapidly, while pyrimidiniums substituted at position 5 (Figure 1, column F) resulted in no active compounds among the halogenated derivatives. Dimethyl imidazoliums (Figure 1, columns H and J) showed limited reactivity, and only the 2-halo-substituted ones were active (**H1+**, **H2+**, **H3+**; I > Br > Cl). Among the other five-membered heterocycles, 2-iodooxazolium (**N3+**) and 2- and 5-bromothiazoliums (**Q2+** and **R2+**, respectively) reacted with GSH, while there was no reaction in the case of 4-iodoisoxazolium (**P3+**). The observed reactivity pattern was consistent with the position of the positive charge in the aromatic ring as observed previously.^18^ The heterocycles having no presumed positive charge on the warhead-substituted carbon [generally *meta* substitution from the heteroatom (Figure 1, columns B, F, J–L, and P)] were mostly not active.

### Electrophilic MiniFrags Identify Novel HDAC8 Binding Sites

**Figure 2:**
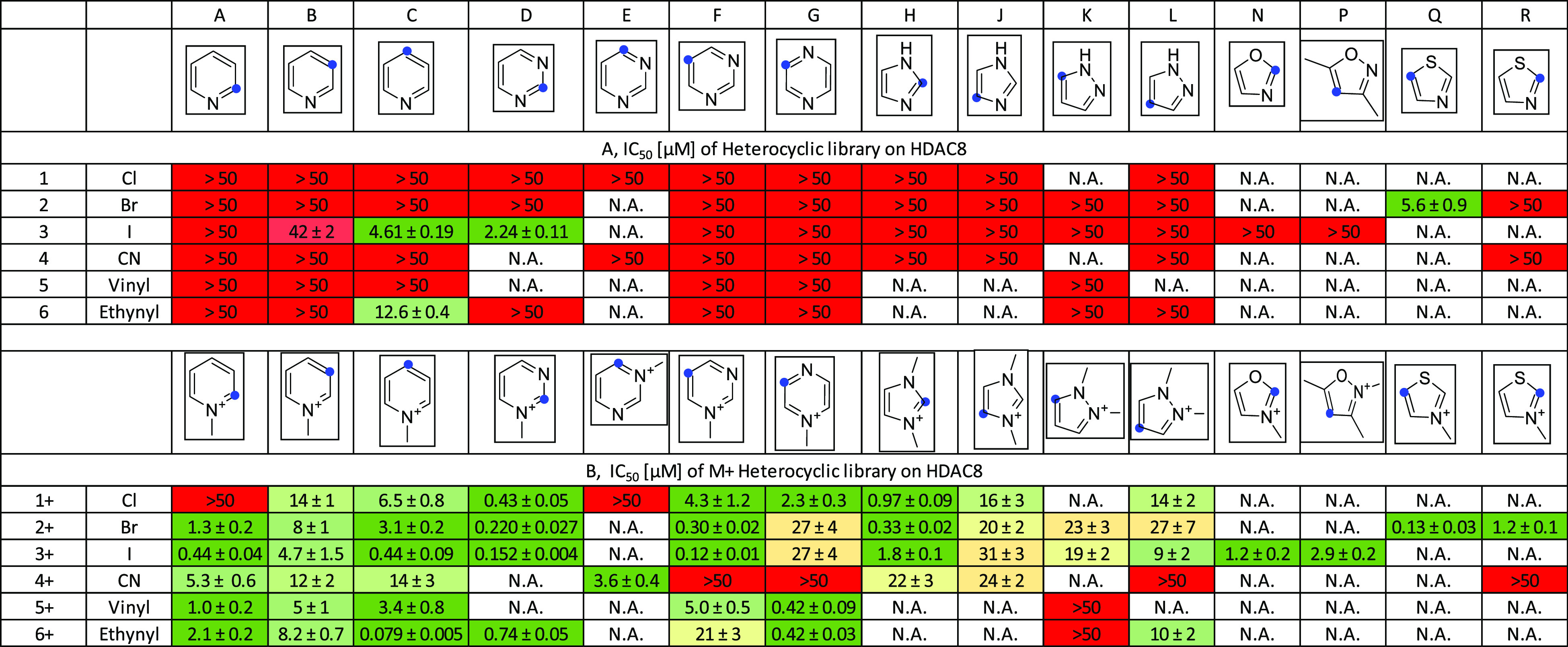
Biochemical assay results obtained testing electrophilic MiniFrags (84 + 58 covalent heterocycles) against HDAC8 represented in IC_50_ values (micromolar, 1 h preincubation, 10 nM HDAC8). Blue dots indicate the position of the warhead. The coloring is in line with the activity, from red (low) through yellow (moderate) to green (high). N.A. stands for “not available”.

The electrophilic MiniFrag library of 84 heterocyclic electrophiles and 58 quaternized analogues was tested in a biochemical HDAC8 assay. From nonmethylated heterocyclic electrophiles, 12 compounds were considered as active (using the threshold IC_50_ < 50 μM) with an average IC_50_ of 25.9 μM (14% hit rate) (Figure 2A). In contrast, the quaternized library provided 54 hits (95% hit rate) with an average IC_50_ of 8.85 μM containing 15 fragments with an IC_50_ of <1 μM (Figure 2B). Head-to-head comparison of nonmethylated and methylated heterocycles showed that the quaternary methylation enhanced the reactivity of all active fragments, increasing the potency to the nanomolar range in many cases. Similar to the surrogate GSH screen, the 2- and 4-pyridiniums (Figure 2, columns A and C) were more active than the 3-pyridiniums (Figure 2, column B), and the cyanide warhead gave the weakest hits (**A4+**, **B4+**, and **C4+**). Among the six-membered heterocycles with two nitrogen atoms, most showed nanomolar activity, except for the halogenated pyraziniums (**G1+**, **G2+**, **G3+**) and 5-ethynyl-pyrimidinium (**F6+**). In general, the increasing number of nitrogen atoms increased the activity in parallel. Among the five-membered heterocycles, the 2-haloimidazoliums (**H1+**, **H2+**, **H3+**), 2-iodooxazolium (**N3+**), 4-iodoisoxazolium (**P3+**), and 2-bromo- (**R2+**) and 5-bromothiazolium (**Q2+**) performed best with low-micromolar IC_50_ values.

Comparing the GSH reactivity and the HDAC8 bioactivity, we can observe that 19 of the 30 GSH-actives gave IC_50_ values of <10 μM, and there were only five inactive compounds. From the 28 GSH-inactives, the IC_50_ was >10 μM in 17 cases and was <5 μM low in 9 cases.

These results showed that the GSH assay for the methylated library was a good indicator for the bioactivity of the reactive compounds, which could be explained with several available cysteine residues on HDAC8, most of which are regulatory. Notably, the surrogate assay results and the protein screening data discussed also showed that the GSH-inactive compounds might also be able to label and inhibit the targeted protein.^14^

Next, the protein labeling of the 10 best-performing methylated fragments (**A3+**, **C3+**, **C6+**, **D1+**, **D2+**, **D3+**, **D6+**, **F2+**, **F3+**, and **G6+**) with IC_50_ values of 77–664 nM was challenged by two orthogonal investigations. First, the activity of the compounds was studied in biochemical assays against mutated HDAC8 proteins (at a protein concentration of 100 nM), where the cysteines were systematically mutated to serines. Second, the compounds were incubated with an entirely reduced HDAC8 followed by tryptic digestion and MS/MS analysis. The results of these parallel investigations showed that although there were privileged cysteines labeled by most of the fragments, the pattern of labeling and IC_50_ values measured on the mutants were different. To rationalize the observed labeling, the reactivity and accessibility of all the available cysteines were investigated using the Cy-preds^34^ and C-PIPE^35^ approaches. These tools characterize the cysteines with several calculated parameters, like p*K*_a_, H-bond contributions (expressed as the p*K*_a_ shift due to H-bonding ability), exposure, hydrophobicity, disulfide-bonding ability, and predicted reactivity. In addition, we used the FTMap methodology to analyze fragment binding hot spots on the surface of HDAC8.^36,37^ The predicted binding hot spots were cross-checked against the proximity of the 10 cysteine residues of HDAC8 (at least one probe atom within the 5 Å radius of any atom of the cysteine) for all of the 18 wild-type Protein Data Bank (PDB) structures that were checked.

The MS/MS investigation (Figures S5–S10) proposed privileged cysteines for labeling. In particular, Cys153 was modified by nine fragments, Cys314 and Cys28 were modified by eight and seven fragments, respectively, Cys244 was modified by six fragments, and Cys275 was modified by five fragments. Cys352 and Cys102 were labeled by only one fragment. However, Cy-Preds and C-PIPE predicted only Cys153 as consistently accessible and reactive, having the lowest p*K*_a_ (5.75 ± 0.28) and largest H-bonding contribution (−3.60 ± 0.21). Cys28, Cys102, Cys131, Cys275, and Cys287 were predicted to be reactive for some but not all of the examined PDB structures. Disulfide bonds were proposed between Cys125 and Cys131 and between Cys244 and Cys287. The most accessible HDAC8 cysteine is Cys352; however, it was labeled by only one fragment that might underlie an equilibrium or kinetically driven selection of privileged Cys residues. Although Cys275 was the second most accessible cysteine in HDAC8, the labeling was accomplished by selective fragments only. Notably, it is possible that a chemical modification at this position is influencing the active site throughout Met274, which is directly involved in substrate binding by forming the surface of the binding channel.^31^ Just like Cys28, Cys314 seems to be a very promising residue, because this cysteine is not involved in any disulfide bond and was labeled by many fragments. Thus, labeling these residues by most of the fragments supports effective follow-ups in this direction. FTMap hot spot analysis showed privileged locations for binding hot spots in the vicinity of Cys131 and Cys153 with frequent occurrences of Cys28, and occasional occurrences are shown for Cys102 and Cys275. This also corresponds with the efficient labeling of Cys153 and Cys28.

**Figure 3:**
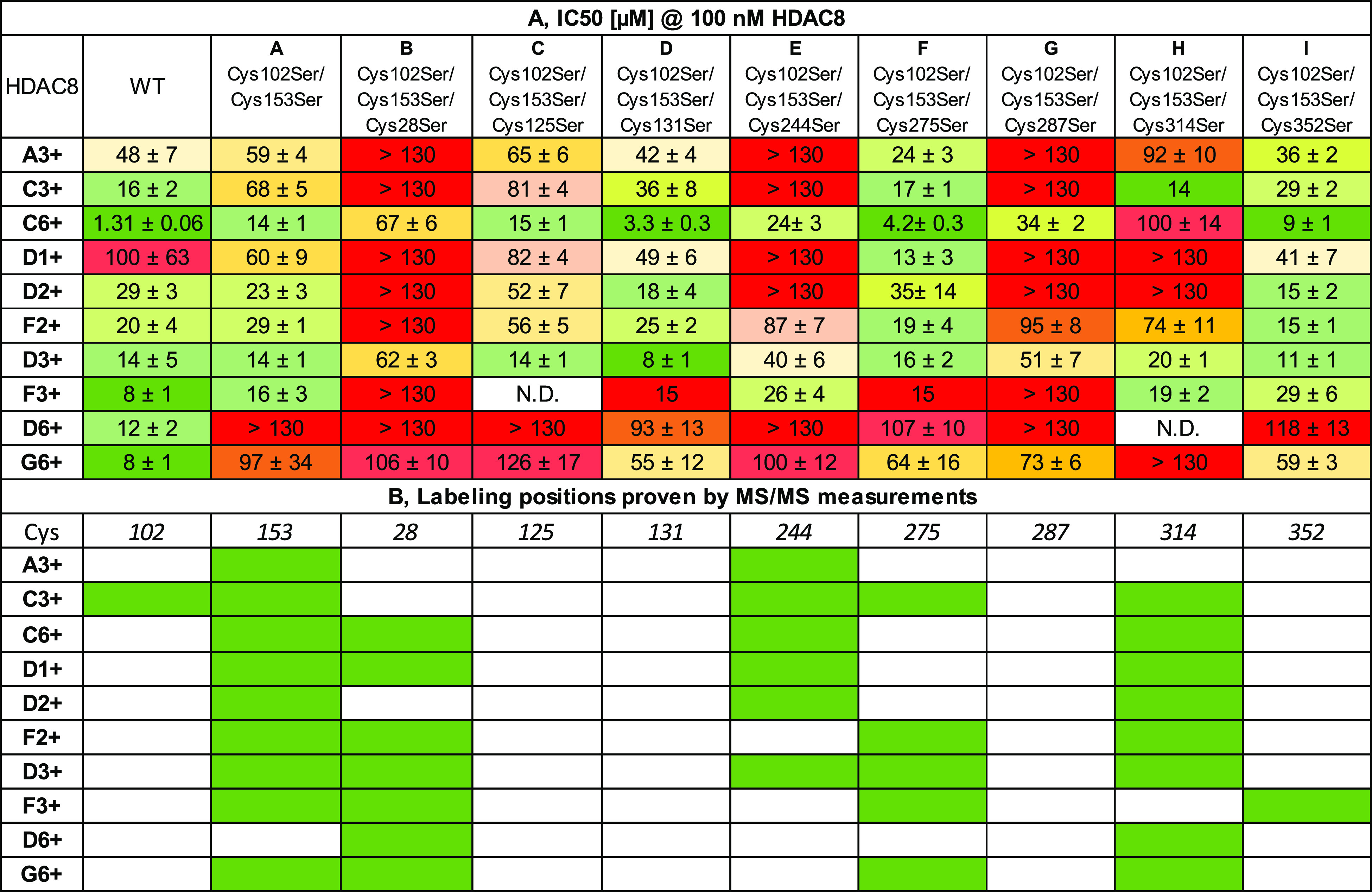
Identification of HDAC8 binding sites by mutational and MS/MS data. (A) Results of the biochemical assay on wild-type (WT) HDAC8 and mutants represented in IC_50_ values (micromolar, 1 h preincubation, 100 nM HDAC8). N.D. stands for “not determined”. (B) MS/MS analysis of labeling of wild-type HDAC8. Green cells stand for labeled cysteines.

We used 11 HDAC8 mutants for the further validation of the labeling patterns. In particular, the selected compounds were tested against two single mutants (Cys102Ser and Cys153Ser), a double mutant (Cys102Ser/Cys153Ser), and eight triple mutants (each cysteine together with Cys102Ser/Cys153Ser). Analyzing the results of the biochemical assays, we could conclude that the single mutations Cys102Ser and Cys153Ser did not result in any significant effect (see Table S1), while the double mutant Cys102Ser/Cys153Ser showed a relevant decrease in potency for five fragments; from those, covalent labeling by three fragments was confirmed (Figure 3, column A). Forming the triple mutants, Cys125Ser (Figure 3, column C), Cys131Ser (Figure 3, column D), Cys275Ser (Figure 3, column F), and Cys352Ser (Figure 3, column I) together with Cys102Ser/Cys153Ser, did not show significant difference from the IC_50_ values measured on the double mutant. In the case of six fragments, the IC_50_ values increased drastically with triple mutant Cys102Ser/Cys153Ser/Cys314Ser (Figure 3, column H), suggesting a significant effect of the Cys314Ser mutation. Notably, five of those fragments labeled Cys314 covalently. Upon mutation of Cys28, Cys244, or Cys287, all fragments lost inhibition or IC_50_ values became 3–20 times higher (Figure 3, column B, E, or G, respectively). These results underlined the significance of Cys28, where the largest difference was observed, and the proximity suggests a similar role for Cys244 and Cys287.

### Electrophilic MiniFrags Are Viable Starting Points for Developing Covalent HDAC8 Inhibitors

We have chosen (*R*)-2-amino-3-(2,4-dichlorophenyl)-1-(1,3-dihydroisoindol-2-yl)propan-1-one (**1**), which is a known HDAC8 binder with a determined crystal structure (PDB entry 3SFH), and proposed that the dichlorophenyl ring could be substituted by a heterocycle to afford Cys153.^38^

Therefore, we first investigated the utility of all MiniFrag hits by molecular modeling; we (i) designed virtual molecules by merging the MiniFrag hits to the isoindoline core of **1** with different linkers and (ii) docked the virtual molecules into the binding site of HDAC8 (PDB entry 3SFH) and compared the resulting poses to the original binding mode of **1**.

**Scheme 2:**
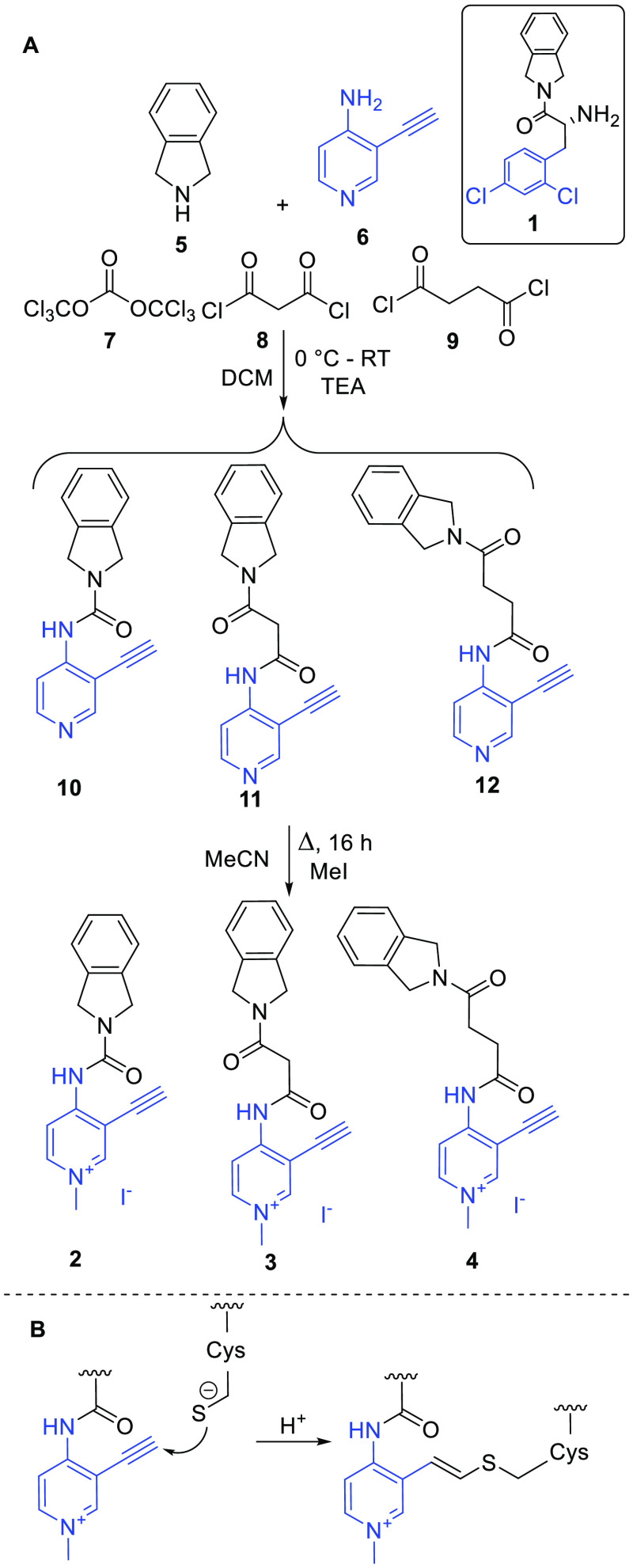
(A) Synthesis of Compounds **2–4** Designed by Merging **1** with **B6+** and (B) Proposed Cysteine Labeling Reaction of Compound **3**

On the basis of the modeling, we have designed three compounds (**2**–**4**) in which **B6+** is connected to the isoindoline with three different linkers (Scheme 2A). We assumed that the acetylene group acts as a Michael acceptor-type covalent warhead reacting with the thiolate of HDAC8.^39,40^

With this approach, we were able to test the effect of the linker length on the biochemical efficacy and turn the reversible inhibitor to irreversible.

Compounds **2–4** were synthesized in the reaction of isoindoline (**5**) and 4-amino-3-ethynylpyridine (**6**) with triphosgene (**7**), malonyl chloride (**8**), and succinyl chloride (**9**), respectively, resulting in nonmethylated compounds **10**–**12**, followed by methylation using MeI (Scheme 2).

**Figure 4:**
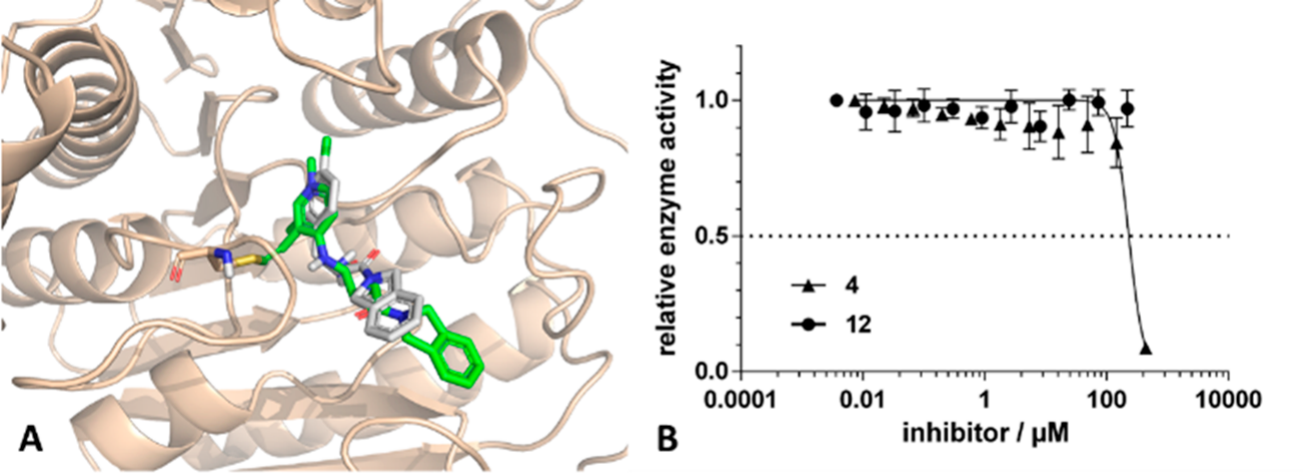
(A) Predicted binding mode of **4** (green molecule) compared to that of **1** (gray molecule) (PDB entry 3SFH). (B) Biochemical assay results of **12** and **4**. The preincubation time was 1 h.

Designed compounds were docked by CovDock to the cavity available in the 3SFH structure forming a covalent bond to Cys153. Compound **4** with the longest linker had an acceptable docking pose; however, the position of the isoindoline amide core was substantially different [atomic root-mean-squared deviation (RMSD) of 2.62 Å] from its placement in the original inhibitor (Figure 4A). In fact, both nonmethylated **12** and methylated **4** showed no activity up to 100 μM in the biochemical assay (Figure 4B).

**Figure 5:**
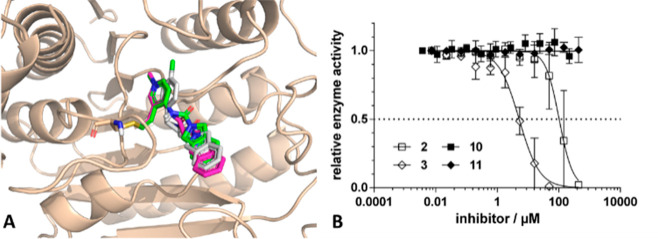
(A) Predicted binding mode of **2** (green molecule) and **3** (magenta molecule) compared to that of **1** (gray molecule) (PDB entry 3SFH). (B) Biochemical assay results of compounds **2**, **3**, **10**, and **11**. The preincubation time was 1 h.

Upon inspection of the inhibitors with medium (**11** and **3**) and short linkers (**10** and **2**), the docked poses showed significantly better agreement in the position of the isoindoline amide core, with RMSD values of 1.08 and 0.60 Å, respectively (Figure 5A).

Notably, in these cases, nonmethylated **10** and **11** were still inactive up to 100 μM, while methylated analogues **2** and **3** had IC_50_ values of 102 and 5.1 μM, respectively (Figure 5B), suggesting that the medium linker (**3**) is the most effective way to couple the two rings (i.e., **1** and **B6+**). The difference in biochemical activity between compounds **3** and **11** suggests the advantageous effect of methylation on the reactivity of the heterocyclic warhead. On the contrary, methylated compounds **2** and **4** were practically inactive, supporting the idea that in addition to the reactivity the appropriate orientation of the warhead, in particular the linker length in this case, also impacts the potency of the covalent inhibitor.

**Figure 6:**
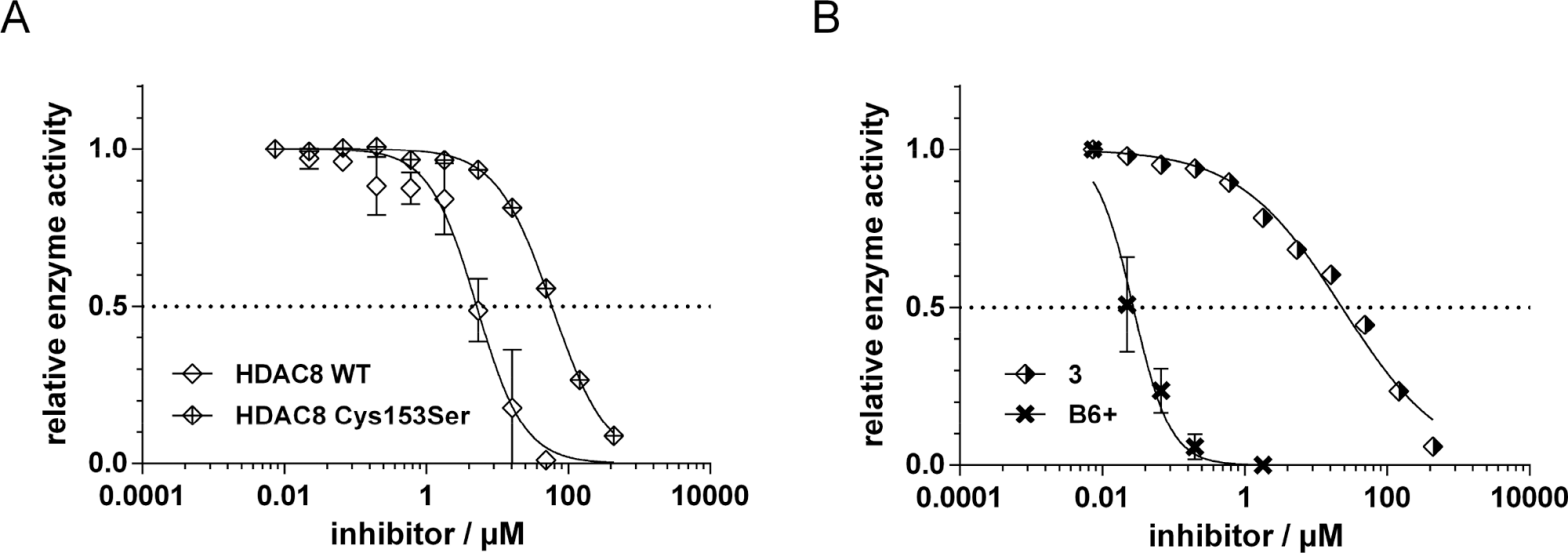
(A) Biochemical activity of **3** on WT HDAC8 and the Cys153Ser mutant. (B) Biochemical activity of **B6+** and **3** on HDAC4. The preincubation time was 1 h.

Next, we investigated the best inhibitor **3** further and proved Cys153 covalent labeling by tryptic digestion and MS/MS (Figure S7). We demonstrated that its covalent binding is irreversible by maintaining inhibition after 2000-fold dilution by overnight dialysis (Figure S11). Moreover, using the HDAC8 Cys153Ser mutant, the biochemical activity decreased 15-fold to 58.8 μM, suggesting that noncovalent binding is still present, but the covalent labeling of Cys153 enhances the activity (Figure 6A). Inspecting the selectivity of class I HDAC8 against class IIa HDAC4 having the conserved Cys153 revealed that **3** slightly prefers HDAC8 (5.1 μM on HDAC8 vs 23.1 μM on HDAC4), while fragment hit **B6+** showed no selectivity (Figure 6B). The efficiency of covalent bond formation resulted from the reversible initial binding, followed by irreversible inactivation. The kinetic parameters of inactivation (*K*_I_ and *k*_inact_) were determined for **B6+** and **3** (Figure S13). The IC_50_ measurements and the corresponding calculations resulted in similar *k*_inact_ values for the two compounds (0.0032 s^–1^ for **B6+** and 0.0051 s^–1^ for **3**) and slightly different *K*_I_ values (0.8 μM for **B6+** and 3.2 μM for **3**). The *k*_inact_/*K*_I_ value for **B6+** was 4006 M^–1^ s^–1^, while for **3**, it was 1566 M^–1^ s^–1^. We have concluded that both the reversible and the irreversible steps in the binding event play a significant role in the observed HDAC8 inhibition.

**Figure 7:**
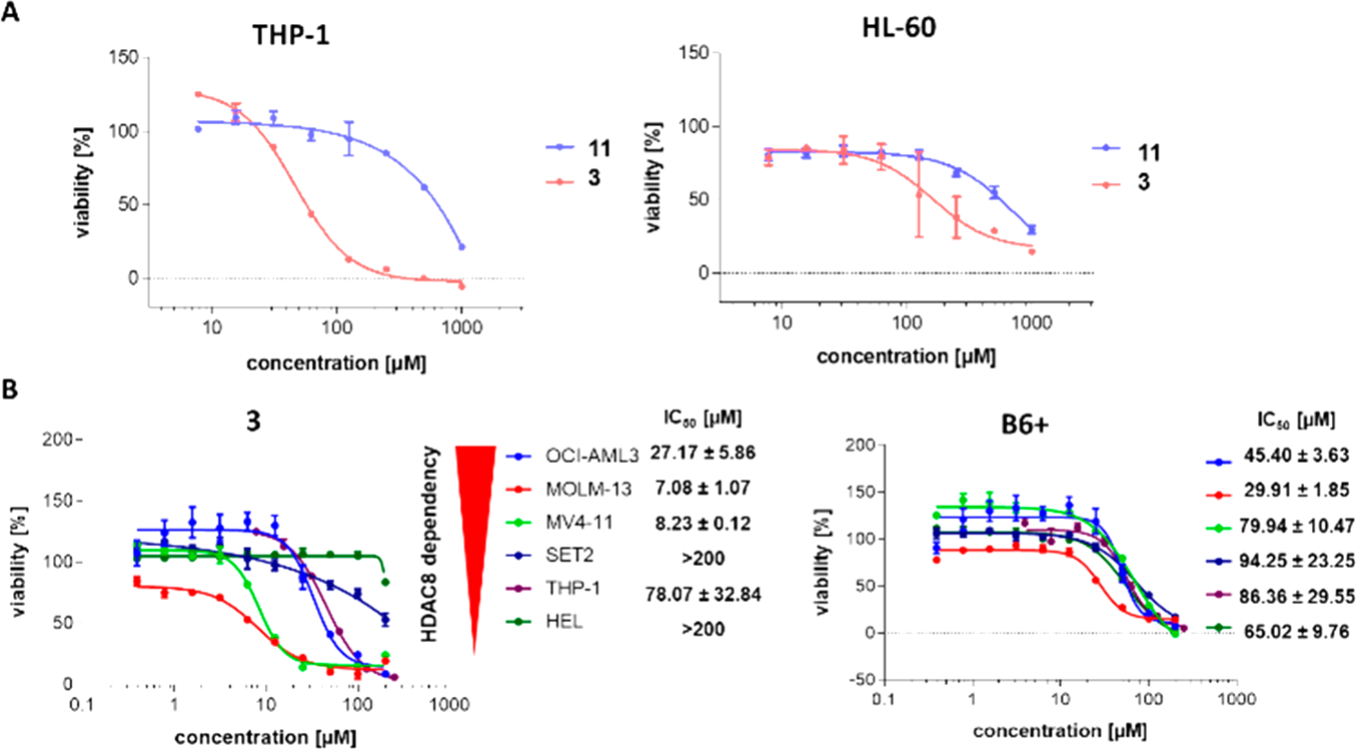
(A) Cytotoxicity assays of THP-1 and HL-60 cell lines treated with the indicated compounds for 72 h. (B) Cytotoxicity assays of compounds **B6+** and **3** on different HDAC8-dependent cell lines. Each cell line was treated with the compound of interest, and the viability was determined using CellTiter-Blue (Promega). Representative dose–response curves are shown, and error bars represent means ± the standard error of the mean (*n* = 2).

Finally, the effect of **3** and its nonmethylated version **11** was tested in HL60 and THP-1 cell lines, known cellular models that are dependent on HDAC8.^41^ The cell viability assay on THP-1 cells confirmed that the methylation resulted in a compound with better cellular activity (IC_50_ values of 46.5 and >500 μM, respectively). The HL60 cell line responded with a higher IC_50_ (161 μM vs >500 μM); however, it still showed a significant difference between the methylated (**3**) and nonmethylated (**11**) compounds (Figure 7A). To compare the selectivity of fragment **B6+** and inhibitor **3**, we have selected cell lines with different HDAC8 dependence based on their behavior toward HDAC8 deletion via CRISPR (depmap.org). Three cell lines were strongly dependent of HDAC8 (MV4–11, MOLM-13, and OCI-AML3), while the other three were HDAC8-independent myeloid leukemia cell lines (HEL, SET-2, and THP-1). The cytotoxicity analysis suggests that HDAC8-dependent cell lines are more sensitive to inhibitor **3**, while for fragment **B6+**, no clear selectivity could be observed (Figure 7B).

Next, to confirm target engagement and functional activity, we chose the OCI-AML3 cell line, and according to the Western blot experiments, already 10 μM **3** or 20–40 μM **B6+** was inducing an increased level of SMC3 acetylation, while not influencing HDAC8 levels, suggesting on-target effects of the two compounds (Figure S14).

## Discussion

**Figure 8:**
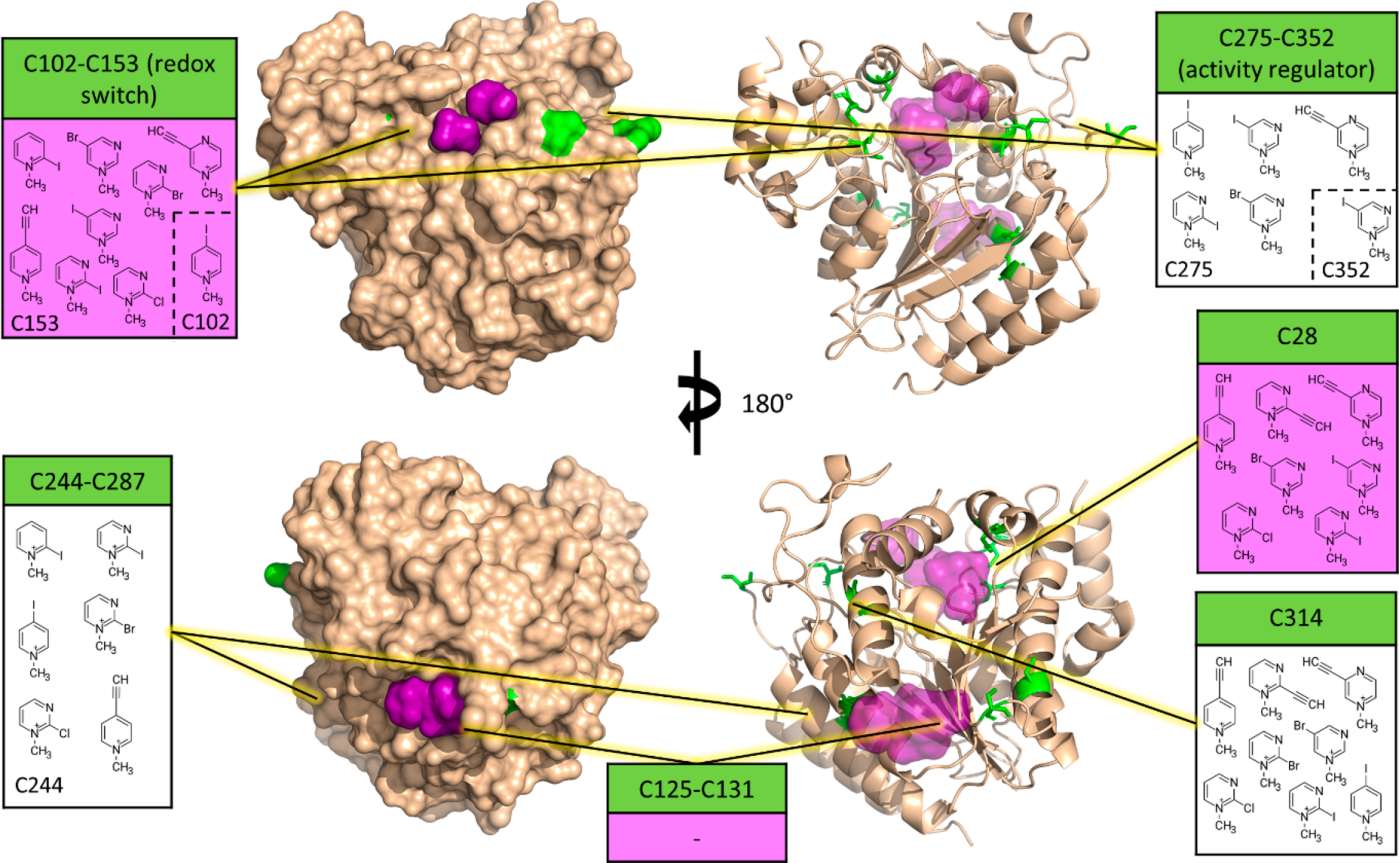
Cysteine residues (green) and predicted fragment binding hot spots (purple) on the surface of HDAC8 (surface and cartoon views from two opposite viewpoints, i.e., top vs bottom images) (PDB entry 3MZ3). The information boxes contain the fragments that were proven to label certain cysteines or disulfide-forming cysteine pairs. Purple areas (and info box color) correspond to binding hot spots. Fragments in dashed brackets have labeled both corresponding cysteines.

HDAC8 is a rather unique protein target when it comes to the design of new targeted covalent inhibitors (TCIs). While usually the question is whether a target has a cysteine available for covalent targeting, HDAC8 possesses no fewer than 10 cysteine residues, resulting in a “confusion of abundance” for medicinal chemists. Here, we have reported several electrophilic MiniFrags that have potently inhibited HDAC8 activity, and by identifying the locations of covalent labeling by MS/MS, we provide a practical overview of the different ways this unique protein can be targeted by TCIs. Figure 8 shows the FTMap^36,37^ predicted binding hot spots successfully labeled by electrophilic MiniFrags.

Of the 10 cysteine residues of HDAC8, eight are involved in disulfide bond formation under physiological conditions. The MS/MS measurements were conducted in a fully reduced state of HDAC8, so theoretically, all cysteines were available for covalent targeting without disulfide formation as a competing process. Nonetheless, several cysteines have stood out in terms of confirmed labeling fragments, while others were left completely unlabeled.

Cysteines 125 and 131 are capable of disulfide formation, and we have recently proposed their role (along with the other disulfide bonds of HDAC8) in redox-based regulation mechanisms of this specific enzyme (C125 and C131 themselves being unique to HDAC8 among its human isoenzymes); however, their importance is yet to be fully understood.^31^ Surprisingly, these residues were not labeled by any of the fragments, despite being located in the vicinity of a fragment binding hot spot.

We have recently established the C275–C352 pair as an allosteric regulator that can decrease enzyme activity by ∼50% upon disulfide bond formation.^31^ The C244–C287 pair is tightly packed against each other, connecting two adjacent α-helices in the vicinity of the Zn binding site, and our results on the C102/C153/C244 and C102/C153/C287 triple mutants hint at their importance in activity regulation (Figure 3). Each of these disulfide-forming pairs was labeled by six fragments, and interestingly, labeling occurred completely and/or almost exclusively on one residue of these pairs: C244 and C275, respectively (the former being unique to HDAC8). Unfortunately, these residues are not ideally located for rational drug design efforts, with the C275–C352 pair being in solvent-exposed and flexible loops and the C244–C287 pair being very close to each other (3.6 Å), thereby presenting strong competition against covalent binders.

Most importantly from the disulfide-forming pairs, there is the main redox switch C102–C153,^30^ being the most abundantly labeled pair of cysteines (nine fragments), although almost exclusively at C153 (except for methyl-pyridinium fragment **C3+**). Therefore, C153 constitutes an almost ideal choice for covalent targeting, (i) being central to enzyme activity, (ii) being located near a binding hot spot, (iii) having the lowest predicted p*K*_a_ value, and (iv) showing the highest reactivity with the sulfenamide inhibitor.^31^ On the contrary, as the main regulator of HDAC activity, it is conserved across all proteins of the HDAC family, which suggests the importance of specific noncovalent interactions in isozyme selectivity.

This strategy has been confirmed by merging originally nonselective fragment hit **B6+** with HDAC8 inhibitor **1** that resulted a novel covalent inhibitor **3** with improved selectivity for class I HDAC8 compared to that for class II HDAC4.

Finally, C28 and C314 were also abundantly labeled with seven and eight fragments, respectively. Of these, C28 in particular seems to be a promising option for targeting HDAC8, being (i) unique to this isozyme, (ii) located at an allosteric regulative domain,^32^ and (iii) located in the vicinity of a fragment binding hot spot. In addition, our results for the C102/C153/C28 triple mutant further highlight its importance for HDAC8 inhibition. More specifically, C28 is adjacent to a small, buried binding site that could be utilized for the optimization of fragment-sized, selective covalent HDAC8 inhibitors. While C314 is also a promising candidate on the basis of the fragment labeling results, the mentioned advantages make C28 a preferred candidate for allosteric targeting.

## Conclusions

The identification of unprecedented binding sites is a challenging task. Biophysical screening of low-molecular weight fragments (MiniFrags) was found to be useful in identifying novel ligand binding pockets; however, weak potencies make the detection of the binding event difficult. Our library of electrophilic MiniFrags offers a unique opportunity to identify tractable binding sites equipped with suitable Cys residues. Fragments bound covalently to the allosteric sites combine the advantages of a covalent mechanism of action with the specificity of allosteric ligands and provide viable starting points for developing covalent allosteric modulators. Here, we have compiled a library of electrophilic MiniFrags consisting of small heterocyclic electrophiles (84 fragments) and their N-quaternized analogues (58 fragments). New derivatives were characterized against GSH in an HPLC/MS-based surrogate assay, demonstrating their enhanced thiol reactivity caused by the methylation of the aromatic nitrogen. Electrophilic MiniFrags screened against HDAC8 provided several hits, including low-nanomolar fragments from the quaternized subset. The biological assay also provided evidence that the methylated heterocycles have consistently greater potencies. Labeling of HDAC8 cysteines was proven by MS/MS studies, and the measurements confirmed different labeling patterns for the heterocycles. Site specific labeling information together with mutational data, theoretical hot spots, and cysteine accessibility and reactivity analyses were used for binding site mapping on HDAC8. Mutating the cysteine residues revealed the influence and functional role of each labeled cysteine. On the basis of these data, we identified Cys28, Cys244, and Cys314 as potential targets for allosteric covalent HDAC8 inhibitors. Finally, starting from a viable fragment hit labeling Cys153 and using a merging strategy with a known noncovalent inhibitor, we identified the first lead-like covalent inhibitor of HDAC8.

## Experimental Section

### General Procedures

All >95% pure chemicals and solvents were purchased from commercial vendors (Sigma-Aldrich, Fluorochem, and Combi-Blocks) and used without further purification. ^1^H NMR and ^13^C NMR spectra were recorded in a DMSO-*d*_6_, CD_3_CN, or D_2_O solution at room temperature on a Varian Unity Inova 500 spectrometer (500 and 125 MHz for ^1^H NMR and ^13^C NMR spectra, respectively), with the deuterium signal of the solvent as the lock. Chemical shifts (δ) and coupling constants (*J*) are given in parts per million and hertz, respectively. HPLC-MS measurements were performed using a Shimadzu LC-MS-2020 device equipped with a Reprospher-100 C18 (5 μm, 100 mm × 3 mm) column and a positive–negative double ion source (DUIS±) with a quadrupole MS analyzer in the range of *m*/*z* 50–1000. The sample was eluted with gradient elution using eluent A (0.1% formic acid in water) and eluent B (0.1% formic acid in acetonitrile). The flow rate was set to 1 mL/min. The initial condition was 0% B eluent, followed by a linear gradient to 100% B eluent by 1 min. From 1 to 3.5 min, 100% B eluent was retained, and from 3.5 to 4.5 min, the initial condition with 5% B eluent was restored and retained until 5 min. The column temperature was kept at room temperature, and the injection volume was 1–10 μL. The purity of the compounds was assessed by HPLC with UV detection at 254 nm; all tested compounds were >95% pure. High-resolution mass spectrometric measurements were performed using a Q-TOF Premier mass spectrometer (Milford, MA) in positive or negative electrospray ionization mode. Reactions were monitored with Merck (Darmstadt, Germany) silica gel 60 F^254^ TLC plates. The column chromatography purifications were performed by using Teledyne ISCO CombiFlash Lumen+ R_f_. All compounds were >95% pure as determined by HPLC analysis.

### Synthetic Procedures

Synthesis and characterization of the MiniFrag library are described in details in refs (15) and (18).

#### Synthesis and Characterization of Compounds **10**–**12** and **2**–**4**

##### General Acylation Protocol

In a round-bottom flask, the corresponding acyl chloride or triphosgene (1 mmol) was stirred in 10 mL of dichloromethane (DCM) under argon at 0 °C. To 5 mL of DCM was slowly added 3-ethynylpyridin-4-amine (1 mmol) together with DIPEA (1.2 mmol). After 1 h in 5 mL of DCM, isoindoline dihydrochloride (1 mmol) was slowly added together with DIPEA (3.6 mmol). The reaction mixture was stirred at room temperature overnight. In case of compounds **11** and **12**, the reaction mixture was washed with 20 mL of water. The organic phase was dried, and the solvent was evaporated. The crude product was purified by preparative HPLC (eluent, acetonitrile/water with 0.1% formic acid). In the case of **10**, the product crashed out of the mixture, and the solid was filtered, washed with 20 mL of water, and dried under air.

##### General Methylation Protocol

Compounds **10**–**12** (0.1 mmol) were stirred in 2 mL of acetonitrile, and 15 μL of iodomethane (0.25 mmol) was added. The reaction mixture was stirred at room temperature overnight. The solvent and the excess of the reagent were evaporated, and the crude product was purified by preparative HPLC (eluent, acetonitrile/water with 0.1% formic acid).

##### *N*-(3-Ethynylpyridin-4-yl)isoindoline-2-carboxamide (**10**)

Yield 110 mg as a yellow solid (42%); ^1^H NMR (500 MHz, DMSO-*d*_6_) δ 8.55 (s, 1H), 8.41 (d, *J* = 5.7 Hz, 1H), 8.14 (d, *J* = 5.9 Hz, 1H), 7.78 (s, 1H), 7.41–7.29 (m, 4H), 4.90–4.75 (m, 5H); ^13^C NMR (126 MHz, DMSO-*d*_6_) δ 152.8, 152.6, 150.4, 147.8, 136.7, 128.0, 123.4, 112.3, 107.8, 91.0, 77.0; HRMS (ESI) (M + H)^+^ calcd for C_16_H_14_N_3_O 264.1136, found 264.1133.

##### *N*-(3-Ethynylpyridin-4-yl)-3-(isoindolin-2-yl)-3-oxopropanamide (**11**)

Yield 25 mg as an orange solid (9%); ^1^H NMR (500 MHz, DMSO-*d*_6_) δ 10.54 (s, 1H), 8.70 (d, *J* = 2.4 Hz, 1H), 8.38 (d, *J* = 1.9 Hz, 1H), 8.21 (t, *J* = 2.2 Hz, 1H), 7.42–7.29 (m, 4H), 4.93 (s, 2H), 4.70 (s, 2H), 4.44 (s, 1H), 3.62 (s, 2H); ^13^C NMR (126 MHz, DMSO-*d*_6_) δ 166.8, 166.1, 147.0, 140.8, 137.0, 136.5, 135.7, 128.6, 128.0, 127.9, 123.5, 123.3, 119.0, 84.5, 80.7, 52.8, 52.3, 44.1; HRMS (ESI) (M + H)^+^ calcd for C_18_H_17_N_3_O_2_ 306.1242, found 306.1240.

##### *N*-(3-Ethynylpyridin-4-yl)-4-(isoindolin-2-yl)-4-oxobutanamide (**12**)

Yield 95 mg as a yellow solid (30%); ^1^H NMR (500 MHz, DMSO-*d*_6_) δ 9.56 (s, 1H), 8.59 (s, 1H), 8.43 (d, *J* = 5.7 Hz, 1H), 8.10 (d, *J* = 5.7 Hz, 1H), 7.41–7.34 (m, 2H), 7.34–7.28 (m, 2H), 4.88 (s, 2H), 4.76 (s, 1H), 4.66 (s, 2H), 2.82 (t, *J* = 6.5 Hz, 2H), 2.73 (t, *J* = 6.5 Hz, 2H); ^13^C NMR (126 MHz, DMSO-*d*_6_) δ 172.6, 170.5, 153.8, 150.4, 146.6, 137.3, 136.7, 127.9, 127.8, 123.5, 123.3, 114.6, 110.0, 109.1, 90.5, 77.1, 52.3, 52.1, 31.9, 29.1; HRMS (ESI) (M + H) ^+^ calcd for C_19_H_18_N_3_O_2_ 320.1399, found 320.1396.

##### 3-Ethynyl-4-(isoindoline-2-carboxamido)-1-methylpyridin-1-ium Iodide (**2**)

Yield 9 mg as a gray solid (84%); ^1^H NMR (500 MHz, DMSO-*d*_6_) δ 9.03 (s, 1H), 8.72 (s, 1H), 8.64 (d, *J* = 7.3 Hz, 1H), 8.50 (d, *J* = 7.3 Hz, 1H), 7.51–7.30 (m, 4H), 5.29 (s, 1H), 5.03 (bs, 2H), 4.87–4.73 (m, 2H), 4.11 (s, 3H); ^13^C NMR (126 MHz, DMSO-*d*_6_) δ 153.0, 151.4, 148.5, 145.7, 136.3, 128.1, 123.4, 113.0, 108.5, 94.3, 73.5, 46.7; HRMS (ESI) (M)^+^ calcd for C_17_H_16_N_3_O 278.1293, found 278.1290.

##### 3-Ethynyl-4-[3-(isoindolin-2-yl)-3-oxopropanamido]-1-methylpyridin-1-ium Iodide (**3**)

Yield 12 mg as an orange solid (40%); ^1^H NMR (500 MHz, DMSO-*d*_6_) δ 11.24 (s, 1H), 9.31 (t, *J* = 1.7 Hz, 1H), 9.01 (d, *J* = 1.5 Hz, 1H), 8.46 (t, *J* = 1.8 Hz, 1H), 7.43–7.28 (m, 5H), 4.97 (s, 1H), 4.94 (s, 2H), 4.70 (s, 2H), 4.35 (s, 3H), 3.73 (s, 2H); ^13^C NMR (126 MHz, DMSO-*d*_6_) δ 167.4, 165.6, 143.2, 138.8, 136.9, 136.4, 136.1, 135.4, 128.1, 128.0, 123.6, 123.3, 122.4, 88.9, 77.1, 52.8, 52.3, 49.3, 44.1; HRMS (ESI) (M)^+^ calcd for C_19_H_18_N_3_O_2_ 320.1399, found 320.1399.

##### 3-Ethynyl-4-[4-(isoindolin-2-yl)-4-oxobutanamido]-1-methylpyridin-1-ium Iodide (**4**)

Yield 8 mg gray solid (95%); ^1^H NMR (500 MHz, DMSO-*d*_6_) δ 10.48 (s, 1H), 9.10 (d, *J* = 1.6 Hz, 1H), 8.74–8.63 (m, 2H), 7.40–7.34 (m, 2H), 7.35–7.29 (m, 2H), 5.21 (s, 1H), 4.89 (s, 2H), 4.66 (s, 2H), 4.15 (s, 3H), 2.96 (dd, *J* = 7.4, 5.2 Hz, 2H), 2.78 (dd, *J* = 7.4, 5.2 Hz, 2H); ^13^C NMR (126 MHz, DMSO-*d*_6_) δ 174.2, 170.4, 151.9, 149.5, 146.0, 137.2, 136.6, 128.0, 127.9, 123.5, 123.3, 115.5, 110.1, 94.3, 73.5, 52.3, 52.2, 47.0, 32.4, 28.9; HRMS (ESI) (M)^+^ calcd for C_20_H_20_N_3_O_2_ 334.1555, found 334.1553.

### Analytics and Biology

#### GSH Assay Based on HPLC-MS

HPLC-MS measurements were performed using a Shimadzu LCMS-2020 device equipped a positive–negative double ion source (DUIS±) and a quadrupole MS analyzer in the range of *m*/*z* 50–1000. The sample was eluted with gradient elution using eluent A (0.1% FA in H_2_O) and eluent B (0.1% FA in ACN). The column temperature was always kept at 30 °C; the injection volume was 20 μL, and the flow rate was set to 1.5 mL/min. For nonmethylated fragments, a Reprospher C18 (5 μm, 100 mm × 3 mm) column was used along with the following gradient. The initial condition was 0% B eluent, followed by a linear gradient to 100% B eluent by 1 min; from 1 to 3.5 min, 100% B eluent was retained. From 3.5 to 4.5 min, theinitial condition with 5% B eluent was restored and retained until 5 min. For methylated fragments, an Inertsil C8 (5 μm, 150 mm × 3 mm) column was used along with the following gradient. The initial condition was 1.5% B eluent, followed by a linear gradient to 30% B eluent by  10 s, and then a linear gradient was used to 95% B eluent by 1.75 min. From 2 min, another gradient was utilized by 30 s to 100% B eluent, and from 2.5 to 2.75 min, the composition of the eluent was set to 5% B and retained until 3.5 min.

For the reactivity and stability assay, a 250 μM solution of the fragment [in PBS buffer (pH 7.4) with 5% acetonitrile] with a 100 μM solution of indoprofen as the internal standard was incubated with or without 5 mM glutathione (providing results of reactivity or stability, respectively). The reaction mixture was analyzed by HPLC-MS sampling after 0, 1, 2, 4, 8, 12, 24, 48, and 72 h. The AUC (area under the curve) values were determined via integration of HPLC or MS chromatograms and then corrected with the internal standard. The fragments’ AUC values were subjected to ordinary least-squares (OLS) linear regression, and to compute the important parameters (kinetic rate constant and half-life time), an Excel sheet was applied. The data are expressed as means of duplicate determinations. The kinetic rate constant for the degradation and corrected GSH reactivity were calculated as follows. The reaction half-life for pseudo-first-order reactions (*t*_1/2_) is ln 2/*k*, where *k* is the reaction rate. In the case of competing reactions (reaction with GSH and degradation), the apparent reaction rate is *k*_app_ = *k*_deg_ + *k*_GSH_. When half-lives are measured experimentally, *t*_1/2(app)_ = ln 2/(*k*_app_) = ln 2/(*k*_deg_ + *k*_GSH_). In our case, the corrected *k*_deg_ and *k*_app_ (regarding blank and GSH-containing samples, respectively) can be calculated by linear regression of the measured kinetic data points. The corrected *k*_GSH_ is calculated as *k*_app_ – *k*_deg_, and finally, the half-life is determined using the equation *t*_1/2_ = ln 2/*k*.

#### Generation, Production, and Purification of HDAC8 Mutant Variants

HDAC8 mutant variants were generated, produced, and further purified as described previously.^32^ Cysteines 28 and 314 were exchanged with serine using the following primer pairs: HD8_C28S_for, TATGTTAGCATGTCTGATAGCCTGGCG; HD8_C28S_rev, CGCCAGGCTATCAGACATGCTAACATA; HD8_C314S_for, AACACCGCGCGTTCTTGGACCTATCTG; HD8_C314S_rev, CAGATAGGTCCAAGAACGCGCGGTGTT.

#### HDAC8 Enzyme-Related Experimental Biochemical Assay against HDAC8, HDAC8 Mutants, and HDAC4

The enzyme activity assay was performed in assay buffer [25 mM Tris-HCl (pH 8.0), 50 mM NaCl, and 0.001% (v/v) Pluronic F-68] in black half-area 96-well microplates (Greiner Bio-One). For the initial screening, 10 nM HDAC8 was preincubated with the indicated compounds at 250 μM for 2 h at 30 °C. For IC_50_ determination, 10 nM HDAC8 (100 nM for the mutational study) and 1 nM HDAC4 were preincubated with a serial dilution of the indicated compounds for 1 h. The reaction was initiated by the addition of 20 μM Boc-Lys(TFA)-AMC (Bachem). After substrate conversion at 30 °C for 15 min for HDAC8 and 1 h for HDAC8 mutants and HDAC4, the reaction was stopped by adding 1.67 μM suberoylanilide trifluoromethylketone (SATFMK). The deacetylated substrate was cleaved with 0.42 mg/mL trypsin to release fluorescent 7-amino-4-methylcoumarin (AMC), which was detected with a microplate reader (PHERAstar FS or BMG LABTECH) with fluorescence excitation at 360 nm and emission at 460 nm. IC_50_ values were calculated by generating dose–response curves in GraphPad Prism 6 and fitting those to a four-parameter logistic model.

#### Determination of the Kinetic Parameters of Inactivation (*K*_I_ and *k*_inact_)

Time-dependent IC_50_ values were obtained using the previously described enzyme activity assay after varying preincubation times. The approach of Krippendorff et al. was implemented in GraphPad Prism 6, and data were fitted accordingly to determine the kinetic parameters of inactivation, *K*_I_ and *k*_inact_; *k*_inact_ is the rate of enzyme inactivation, and *K*_I_ is the inhibitor concentration that results in half of the maximal rate.^42^

#### Cell Viability Assay

All cell lines were purchased from DSMZ (Braunschweig, Germany). The cell lines were regularly tested to exclude mycoplasma contamination and authenticated. Cell lines were grown at 37 °C and 5% CO_2_ in RPMI 1640 medium (Gibco, Thermo Fisher Scientific). Media were supplemented with 10% fetal calf serum (FCS), 10 units/mL penicillin, 10 μg/mL streptomycin, and 2 mM l-glutamine (all Gibco, Thermo Fisher Scientific).

To determine the IC_50_ of the selected compounds on the cell lines, the CellTiter-Blue cell viability assay (Promega) was performed. For this, cells were seeded in 96-well flat-bottom plates at a cell density of 10 000 cells/well. Cells were treated in triplicate with the compound of interest at various concentrations or with 10 μM Bortezomib (S1013; Selleck Chemicals, Houston, TX), as a positive control. The cell viability of treated cell lines was measured using CellTiter-Blue after incubation for 72 h. Plates were measured using a GloMax plate reader (Promega), and IC_50_ values were determined by nonlinear regression using GraphPad Prism version 9.1.1 (GraphPad Software, Inc.), and the data are reported as mean values ± the standard error of the mean.

#### Immunoblot Analysis

Cells (2 ×10^6^) were seeded in 2 mL of medium in a six-well plate (Greiner) and treated with the desired concentration of the compounds. Cells were incubated at 37 °C for 72 h and then lysed in whole cell extract buffer [20 mM HEPES (pH 7.9), 20% glycerol, 50 mM KCl, 1 mM EDTA, 1 mM DTT (Sigma-Aldrich), 400 mM NaCl, 5 μg/mL leupeptin (Sigma-Aldrich), 5 mM β-glycerophosphate, 1 mM PMSF (Sigma-Aldrich), 5 μg/mL aprotinine (Sigma-Aldrich), 10 mM NaF, and 5 mM Na_3_VO_4_]. Protein concentrations were determined by the Bradford protein assay. Thirty micrograms of cell lysates per treatment was fractionated on sodium dodecyl sulfate–polyacrylamide gels and transferred to nitrocellulose membranes (Cytiva). Then, 5% BSA in TBS-T was used for blocking, and antibodies (ac-SMC3, HDAC8) were diluted in TBS-T. Equal loading was confirmed by probing the same membranes with a specific antibody for human ACTIN (1:1000, sc-47778).

#### Labeling and Tryptic Digestion of HDAC8

The covalent labeling procedure was conducted as described previously with slight modifications.^31^ First, 25 μM HDAC8 was treated with 250 μM covalent probes for 1 h at 30 °C in the assay buffer described above. The protein was then precipitated by the addition of 10% TCA and then centrifuged at 18000*g* for 15 min. The supernatant was removed, and the dry pellet was diluted in buffer [50 mM NH_4_HCO_3_ (pH 7.8)]. After the fragment labeling was completed, 50 μL of the sample and 10 μL of a 0.2% (w/v) RapiGest SF (Waters, Milford, MA) solution buffered with 50 mM ammonium bicarbonate were mixed (pH 7.8), 3.3 μL of 45 mM DTT in 100 mM NH_4_HCO_3_ was added to reduce artificial oxidized cysteine residues, and the mixture was kept at 37.5 °C for 30 min. After the sample was cooled to room temperature, reduction was quenched, and nascent thiols were alkylated by adding 4.16 μL of 100 mM iodoacetamide in 100 mM NH_4_HCO_3_. Samples were placed in the dark at room temperature for 30 min. The reduced and alkylated protein was then digested with 10 μL (1 mg/mL) of trypsin (the enzyme:protein ratio was 1:10) (Sigma, St. Louis, MO). The sample was incubated at 37 °C overnight. To degrade the surfactant, 7 μL of a formic acid (500 mM) solution was added to the digested protein sample, a final concentration of 40 mM (pH ≈2) was obtained, and the mixture was further incubated at 37 °C for 45 min. For LC-MS analysis, the acid-treated sample was centrifuged for 5 min at 13 000 rpm and the supernatant was pipetted into a microvial.

For procedure A, an AB Sciex 6500 QTRAP hybrid triple quadruple linear ion trap mass spectrometer, equipped with a Turbo V ion source in electrospray mode and an Agilent 1100 Binary Pump HPLC system (Agilent Technologies, Waldbronn, Germany) consisting of an autosampler, was used for LC-MS/MS analysis. Data acquisition and processing were performed using Analyst version 1.6.2 (AB Sciex Instruments). Chromatographic separation was achieved by using the Discovery BIO Wide Pore C-18-5 column (250 mm × 2.1 mm, 5 μm, 300 Å). The sample was eluted with a gradient of solvent A (0.1% formic acid in water) and solvent B (0.1% formic acid in ACN). The flow rate was set to 0.2 mL min^–1^. The initial process for separation was as follows: 5% B for 7 min, followed by a linear gradient to 90% B by 53 min, 90% B from 60 to 64 min, and from 64 to 65 min back to the initial condition with 5% eluent B retained for 10 min. The injection volume was 10 μL. An information-dependent acquisiton (IDA) LC-MS/MS experiment was used to identify the modified tryptic peptide fragments. An enhanced MS scan (EMS) was used as a survey scan, and an enhanced product ion scan (EPI) was the dependent scan. Precursor ion selection criteria: ions greater than *m*/*z* 400, which exceeds 106 counts, exclude former target ions for 30 s after two occurrence(s). The scan rates in both survey and dependent scans were 1000 Da/s. Nitrogen was used as the nebulizer gas (GS1), heater gas (GS2), and curtain gas with the optimum values set at 50, 40, and 40 (arbitrary units), respectively. The source temperature was 350 °C, and the ion spray voltage was set at 5000 V. The declustering potential value was set to 150 V. The collision energy in EPI experiments was set to rolling collision energy mode, where the actual value was set on the basis of the mass and charge state of the selected ion. GPMAW version 4.2 was used to analyze the large number of MS-MS spectra and identify the modified tryptically digested peptides.

For procedure B, to obtain more precise information about the structure, samples were further analyzed by a Triple TOF 5600+ hybrid Quadrupole-TOF LC/MS/MS system (Sciex) equipped with a DuoSpray IonSource coupled with a Shimadzu Prominence LC20 UFLC system consisting of a quaternary pump, an autosampler, and a thermostated column compartment. Data were acquired and processed using Analyst TF version 1.7.1 (AB Sciex Instruments). Chromatographic separation was achieved on the Discovery BIO Wide Pore C-18-5 (250 mm × 2.1 mm, 5 μm, 300 Å) HPLC column. The sample was eluted in gradient elution mode using solvent A (0.1% formic acid in water) and solvent B (0.1% formic acid in ACN). The initial condition was as follows: 5% B for 7 min, followed by a linear gradient to 90% B by 48 min, 90% B from 55 to 63 min, and from 63 to 65 min back to the initial condition with 5% eluent B and retained for 10 min. The flow rate was set to 0.2 mL/min. The column temperature was 50 °C, and the injection volume was 10 μL. Nitrogen was used as the nebulizer gas (GS1), heater gas (GS2), and curtain gas with the optimum values set at 35, 35, and 35 (arbitrary units), respectively. The source temperature was 350 °C, and the spray voltage was set to 5500 V. Advanced information dependent acquisition (IDA) mode was used on the TripleTOF 5600+ system to obtain MS/MS spectra on the four most abundant parent ions present in the TOF survey scan. In IDA LC-MS/MS experiment, the mass spectra and tandem mass spectra were recorded in “high-sensitivity” mode with a resolution of ∼35 000 full width at half-maximum. In the first period (positive TOF MS mode), the data were acquired in the mass range of *m*/*z* 300–2500, with an accumulation time of 0.25 s. The declustering potential value was set to 60 V. The intensity threshold for precursor ion selection in the TOF survey scan mode was 1000 cps. In the MS2 experiment (product ion scan mode), the mass range was *m*/*z* 50–2000, with an accumulation time of 0.1 s. Peak View Software version 2.2 (Sciex, Redwood City, CA) was used to assign and evaluate the peaks in the MS/MS spectra.

Notably, the sequence of the digested protein samples starts with an additional “H”; therefore, the number of each amino acid is shifted by one. For example, Cys28 is the 29th amino acid in the sequence.

#### Computational Methods

The FTMap method distributes small organic probe molecules of varying size, shape, and polarity on a dense grid defined on the macromolecule surface, finds the most favorable positions for each probe type, performs local energy minimization allowing for probe flexibility, and then clusters the probes and ranks the clusters on the basis of their average energy (current list of probes: ethanol, isopropanol, isobutanol, acetone, acetaldehyde, dimethyl ether, cyclohexane, ethane, acetonitrile, urea, methylamine, phenol, benzaldehyde, benzene, acetamide, and *N*,*N*-dimethylformamide). The 2000 lowest-energy poses for each probe are energy minimized using the CHARMM potential^43^ with the analytic continuum electrostatic (ACE) model^44^ to account for electrostatics and solvation and clustered with a 4 Å radius, starting with the lowest-energy structure. Regions that bind multiple probe clusters are defined as the predicted binding hot spots, which are finally ranked on the basis of the number of different probe clusters they bind. Here, we have cross-checked the predicted binding hot spots against the proximity of the 10 cysteine residues of HDAC8 (at least one probe atom within the 5 Å radius of any atom of the cysteine), and the best (lowest) hot spot ranks were collected for each cysteine residue for all of the 18 wild-type PDB structures that were checked (3RQD, 3SFF, 3F0R, 5FCW, 3SFH, 3F07, 3MZ3, 2V5W, 2V5X, 1VKG, 1W22, 1T69, 1T67, 1T64, 6ODC, 6ODB, 6ODA, and 5VI6).

For a quick assessment of the availability of the cysteine residues for covalent targeting, the CyPreds^33^ [for nine crystal structures (3RQD, 3SFF, 3SFH, 3F07, 3F0R, 3MZ3, 2V5W, 2V5X, and 5FCW) giving very similar results] and CPIPE^34^ [for four crystal structures (3RQD, 3SFF, 3F0R, and 5FCW) giving very similar results] Web servers were used. To that end, the Web servers estimate the accessibility and reactivity of cysteine residues. Briefly, they employ a consensus of multiple approaches for predicting cysteine reactivity, based on sequence profiling, as well as the evaluation of p*K*_a_ values, H-bond contribution terms, and various other descriptors. Classification of the cysteines as reactive/nonreactive is carried out by a simple decision tree, based on the calculated parameters.

LUMO levels were computed using Gaussian 16 applying structure optimization and frequency calculations at the m062x/6-31G(d,p) level of theory (for iodine, the lanl2dz basis set was applied), considering the implicit solvent effect of water (SMD).^45−48^

## Supporting Information Available

Molecular formula strings, residual activity data, dose–response curves, MS/MS spectra, experimental procedures, and compound characterization (PDF)

## Abbreviations

HDAC: histone deacetylase
GSH: glutathione
MS: mass spectrometry
HPLC: high-performance liquid chromatography
NMR: nuclear magnetic resonance
TCI: targeted covalent inhibitor
MurA: UDP-*N*-acetylglucosamine enolpyruvyl transferase
Cys: cysteine
Ser: serine
Met: methionine
PDB: Protein Data Bank
RMSD: root-mean-square deviation
QTOF: quadrupole time-of-flight
DMSO: dimethyl sulfoxide
AUC: area under the curve
IC: inhibitory concentration
TLC: thin layer chromatography
