# Supplementary material for: Electrophilic MiniFrags Revealed Unprecedented Binding Sites for Covalent HDAC8 Inhibitors
Source: J Med Chem. 2023 Dec 19;67(1):572–85. doi: 10.1021/acs.jmedchem.3c01779 (PMC10788917; doi:10.1021/acs.jmedchem.3c01779)
Supplement: Supplementary file 1 — jm3c01779_si_001.pdf [file jm3c01779_si_001.pdf]

Supplementary Information

Electrophilic MiniFrags revealed unprecedented binding sites for covalent HDAC8 inhibitors

Aaron B. Keeley†[a,b,c], Aleksandra Kopranovic†[d], Vincenzo Di Lorenzo†[a,b,c], Péter Ábrányi-Balogh†[a,b,c], Niklas Jänsch[d], Linh N. Lai[d], László Petri[a,b,c], Zoltán Orgován[a,b,c], Daniel Pölöske[e], Anna Orlova[e], András György Németh[a,b,c], Charlotte Desczyk[d], Tímea Imre[a,f], Dávid Bajusz[a,b,c], Richard Moriggl[e], Franz-Josef Meyer-Almes[d] and György M. Keserű[a,b,c]**

[a]Medicinal Chemistry Research Group, Research Centre for Natural Sciences, Magyar tudósok krt 2, H-1117 Budapest, Hungary.

[b]Department of Organic Chemistry and Technology, Faculty of Chemical Technology and Biotechnology, Budapest University of Technology and Economics, Műegyetem rkp. 3., H-1111 Budapest, Hungary

[c]National Laboratory for Drug Research and Development, H-1117 Budapest, Hungary

[d]Department of Chemical Engineering and Biotechnology, University of Applied Sciences Darmstadt, Haardtring 100, 64295 Darmstadt, Germany.

[e]Institute of Animal Breeding and Genetics, University of Veterinary Medicine, 1210 Vienna, Austria.

[f]MS Metabolomics Research Group, Research Centre for Natural Sciences, Magyar tudósok krt 2, H-1117 Budapest, Hungary

Corresponding Author

*keseru.gyorgy@ttk.hu (G.M.K., lead contact), franz-josef.meyer-almes@h-da.de (F.-J. M.-A.)

Table of Contents

Supplementary Figure S1. Thiol reactivity table for heterocyclic electrophiles. 3

Supplementary Figure S2. Dose-response curves for the determination of IC_{50}-values of HDAC8 biochemical assay with the heterocyclic MiniFrags. 4

Supplementary Figure S3. Dose-response curves for the determination of IC_{50}-values on 10 nM HDAC8 and active site mutants with the heterocyclic MiniFrags. 5

Supplementary Figure S4. Dose-response curves for the determination of IC_{50}-values on 100 nM HDAC8 mutants with the heterocyclic MiniFrags. 5

Supplementary Figure S5. The MSMS spectrum and annotation of **C6+**, **D1+**, **F2+**, **D3+**, **F3+**, **D6+**, **G6+** modified HDAC8 peptide [**1-34**] 6

Supplementary Figure S6. The MSMS spectrum and annotation of **C3+** modified HDAC8 peptide [**33-83**] 7

Supplementary Figure S7. The MSMS spectrum and annotation of **A3+**, **C3+**, **C6+**, **D1+**, **D2+**, **F2+**, **D3+**, **F3+**, **G6+** modified HDAC8 peptide [**148-165**] 7

Supplementary Figure S8. The MSMS spectrum and annotation of **A3+**, **C3+**, **C6+**, **D1+**, **D2+**, **D3+** modified HDAC8 peptide [**241-250**] 9
Supplementary Figure S9. The MSMS spectrum and annotation of **C3+**, **F2+**, **D3+**, **F3+**, **G6+** modified HDAC8 peptide [**260-287**] 10
Supplementary Figure S10. The MSMS spectrum and annotation of **F3+** modified HDAC8 peptide [**327-357**] 11
Supplementary Figure S11. Results of the dilution experiments proving the irreversible covalent labeling for compounds 3 and B6+ 11
Supplementary Figure S12. MS Chromatogram for showing the labelling of GSH with **F4+** 12
11
Supplementary Figure S13. Results of time dependent IC_{50} measurements and K_I, k_{inact}, k_{inact}/K_I calculations 13
Supplementary Figure S14. Results of the western blot after 72 h treatment of OCI-AML3 cells 14
NMR spectra and HPLC-MS measurements of lead compounds 2-4 and 10-12 14
Supplementary Tables S1, S2, S3 Zipped Excel files 25

Supplementary Figure S1. Thiol reactivity table for heterocyclic electrophiles. Blue dots indicate the position of the warhead. Colouring is in line with the activity, as the darker green colour refers for higher reaction rate and lower half life. Compounds labelled by italic showed parallel reaction with the assay buffer. N.A. stands for “Not available”.

		A	B	C	D	E	F	G	H	[illegible]	K	L	N	P	Q	R
		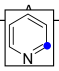	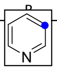	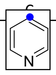	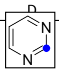	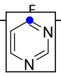	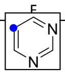	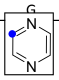	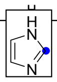	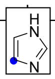	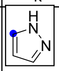	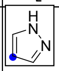	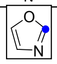	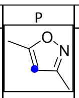	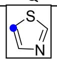	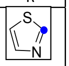
$t_{1/2}$(h) of M+ fragment (250 uM) with GSH (5 mM)																
1	Cl	>48	>48	>48	>48	>48	>48	>48	>48	>48	N.A.	>48	N.A.	N.A.	N.A.	N.A.
2	Br	35.20	>48	>48	>48	N.A.	>48	>48	>48	>48	>48	>48	N.A.	N.A.	>48	>48
3	I	>48	>48	>48	>48	N.A.	>48	>48	0.16	>48	>48	>48	0.23	>48	N.A.	N.A.
4	CN	>48	40.90	0.79	2.00	40.50	>48	21.90	>48	>48	N.A.	>48	N.A.	N.A.	N.A.	8.20
5	Vinyl	1.00	>48	0.26	>48	N.A.	>48	18.50	N.A.	N.A.	>48	N.A.	N.A.	N.A.	N.A.	N.A.
6	Ethynyl	>48	>48	2.40	43.80	N.A.	>48	>48	N.A.	N.A.	5.70	2.20	N.A.	N.A.	N.A.	N.A.

Supplementary Figure S2. Dose-response curves for the determination of IC_{50}-values of HDAC8 biochemical assay with the heterocyclic MiniFrags.

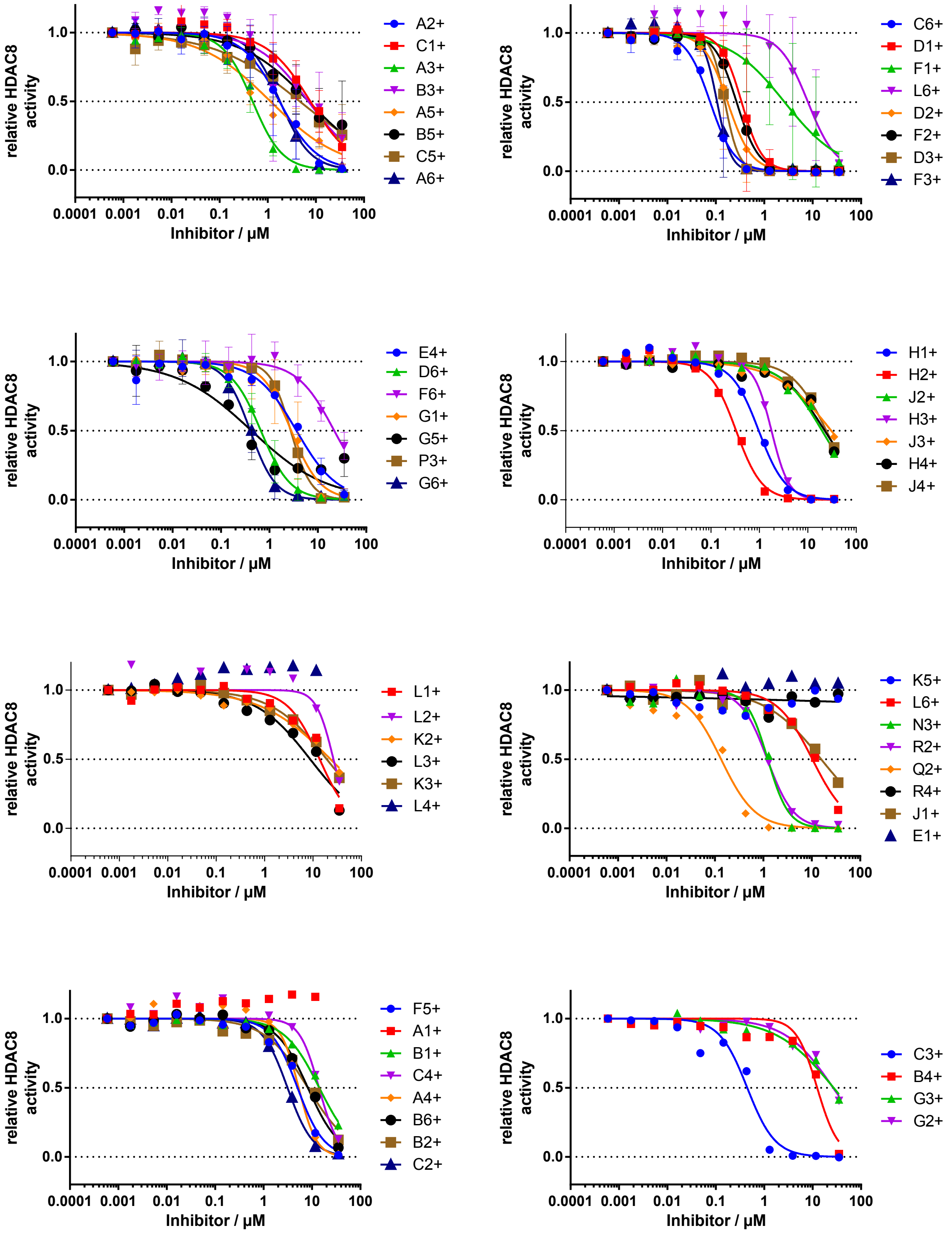

Supplementary Figure S3. Dose-response curves for the determination of IC_{50}-values on 10 nM HDAC8 and active site mutants with the heterocyclic MiniFrags.

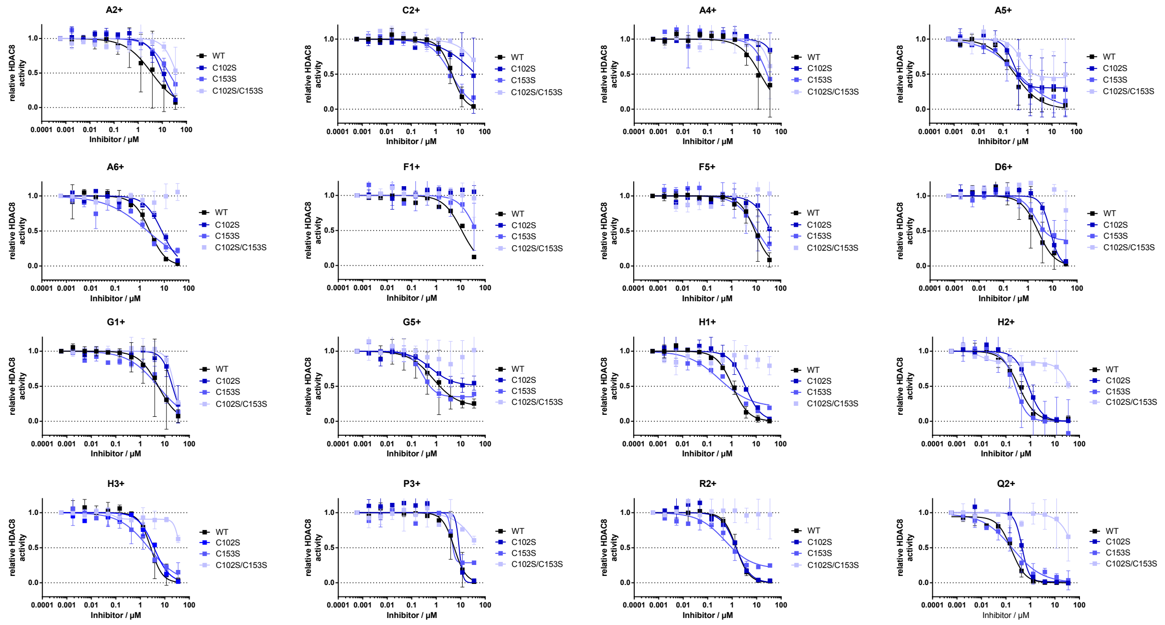

Supplementary Figure S4. Dose-response curves for the determination of IC_{50}-values on 100 nM HDAC8 mutants with the heterocyclic MiniFrags.

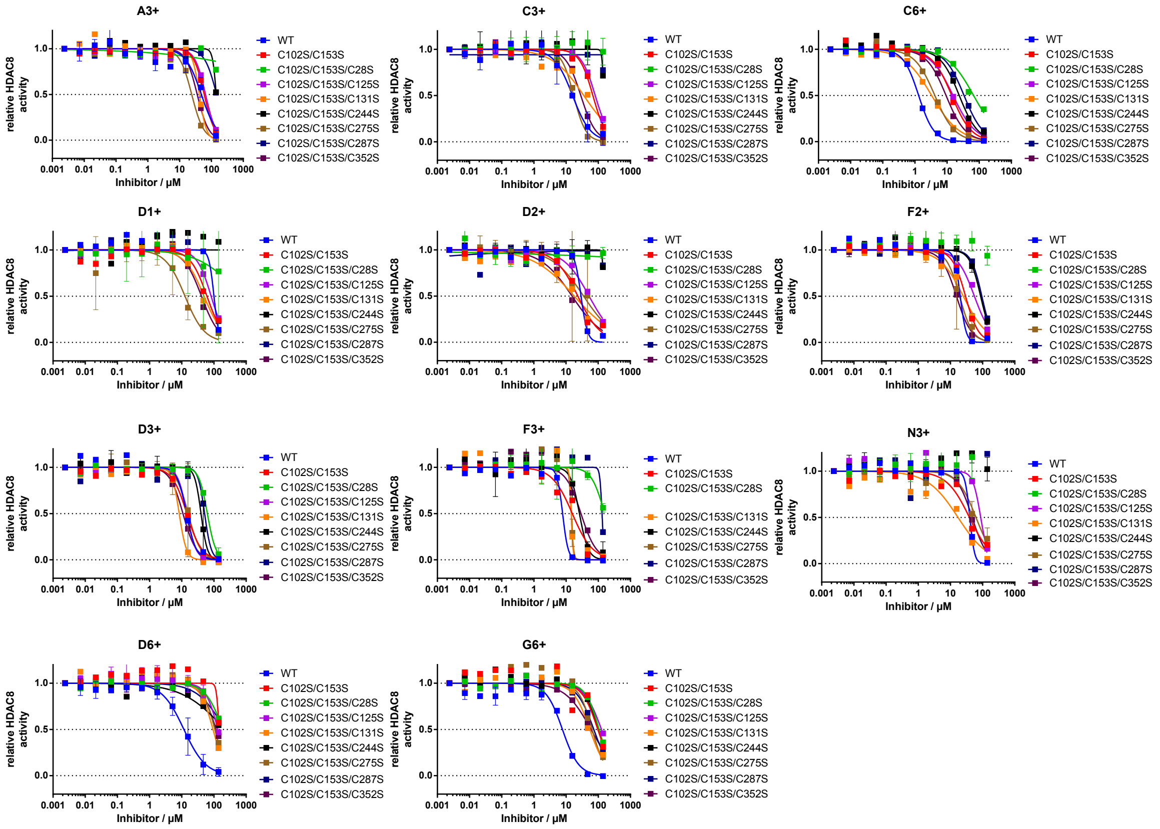

Supplementary Figure S5. The MSMS spectrum and annotation of **C6+**, **D1+**, **F2+**, **D3+**, **F3+**, **D6+**, **G6+** modified HDAC8 peptide [**1-34**]

C6+

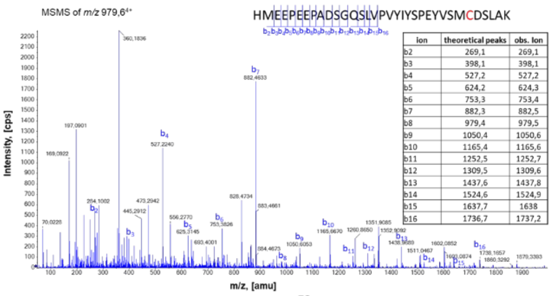

D1+

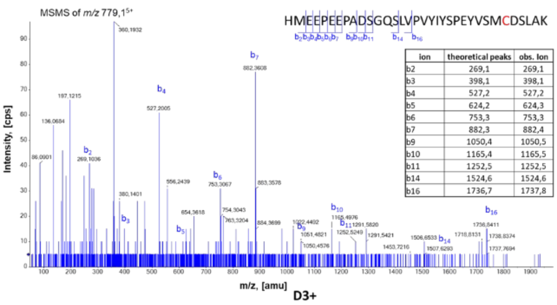

F2+

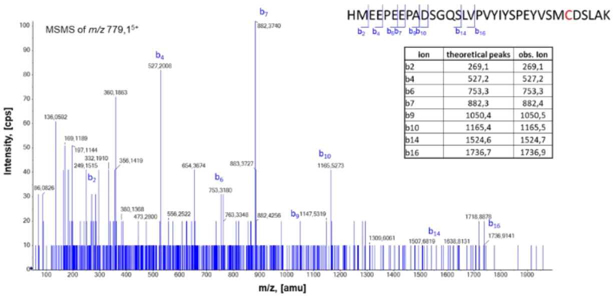

D3+

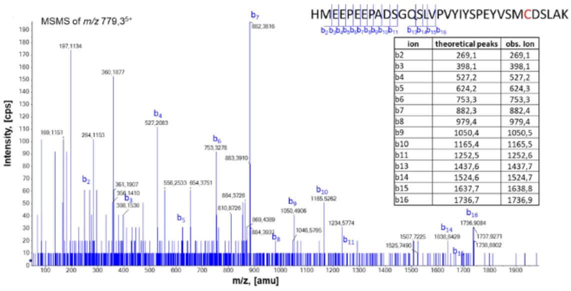

F3+

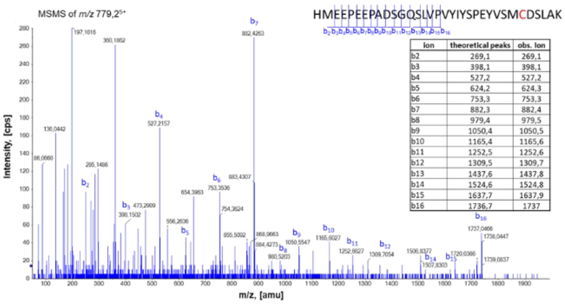

D6+

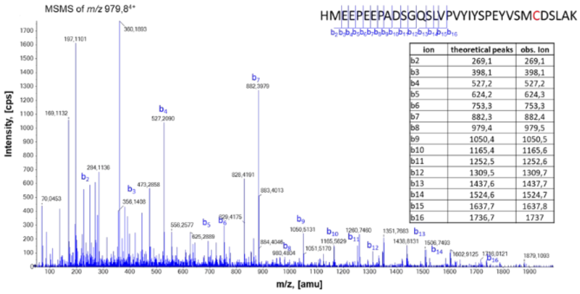

G6+

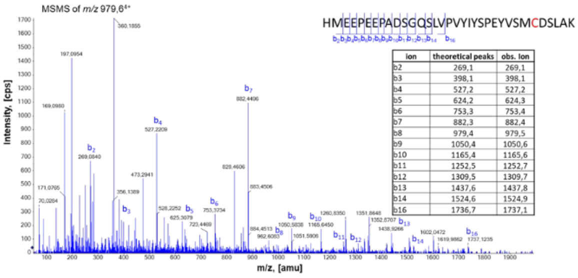

Supplementary Figure S6. The MSMS spectrum and annotation of **C3+** modified HDAC8 peptide [**33-83**]

C3+

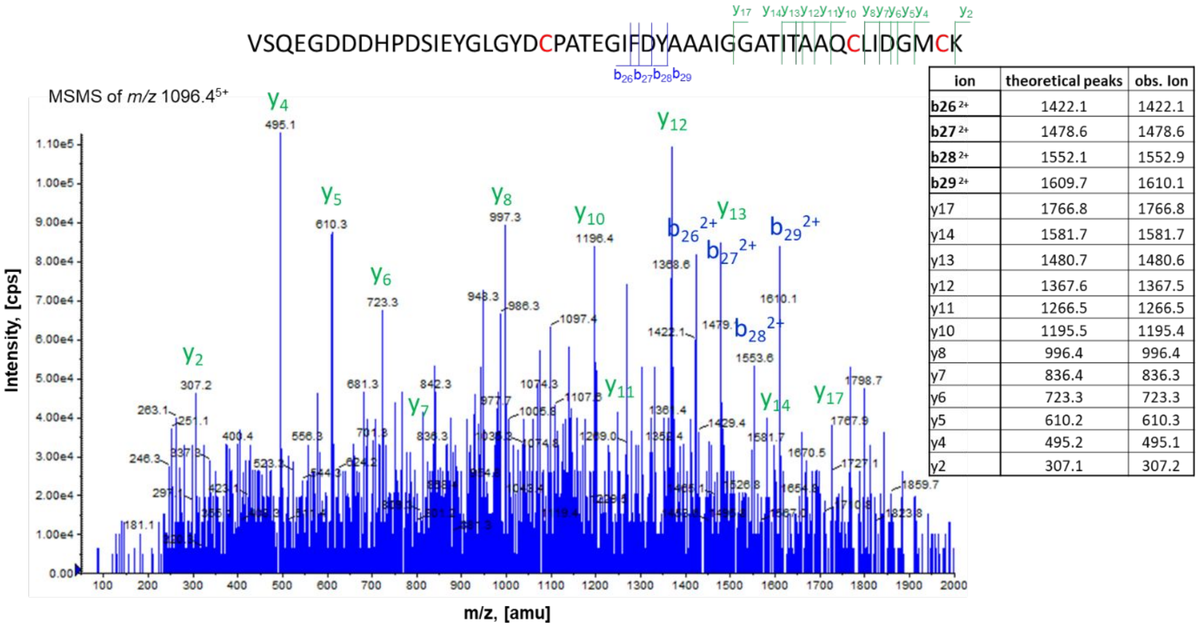

ion	theoretical peaks	obs. Ion
b26 $^{2+}$	1422.1	1422.1
b27 $^{2+}$	1478.6	1478.6
b28 $^{2+}$	1552.1	1552.9
b29 $^{2+}$	1609.7	1610.1
y17	1766.8	1766.8
y14	1581.7	1581.7
y13	1480.7	1480.6
y12	1367.6	1367.5
y11	1266.5	1266.5
y10	1195.5	1195.4
y8	996.4	996.4
y7	836.4	836.3
y6	723.3	723.3
y5	610.2	610.3
y4	495.2	495.1
y2	307.1	307.2

Supplementary Figure S7. The MSMS spectrum and annotation of **A3+**, **B6+**, **C3+**, **C6+**, **D1+**, **D2+**, **F2+**, **D3+**, **F3+**, **G6+, 3** modified HDAC8 peptide [**148-165**]

A3+

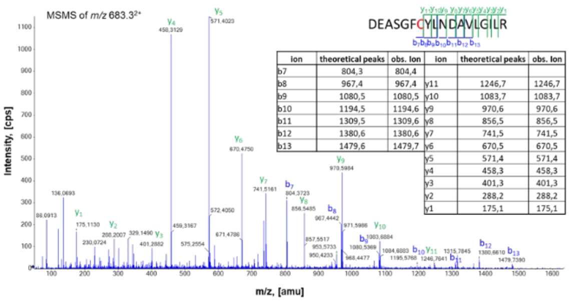

ion	theoretical peaks	obs. Ion	ion	theoretical peaks	obs. Ion
b7	804,3	804,4			
b8	967,4	967,4	y11	1246,7	1246,7
b9	1080,5	1080,5	y10	1083,7	1083,7
b10	1194,5	1194,6	y9	970,6	970,6
b11	1309,5	1309,6	y8	856,5	856,5
b12	1380,6	1380,6	y7	741,5	741,5
b13	1479,6	1479,7	y6	670,5	670,5
			y5	571,4	571,4
			y4	458,3	458,3
			y3	401,3	401,3
			y2	288,2	288,2
			y1	175,1	175,1

C3+

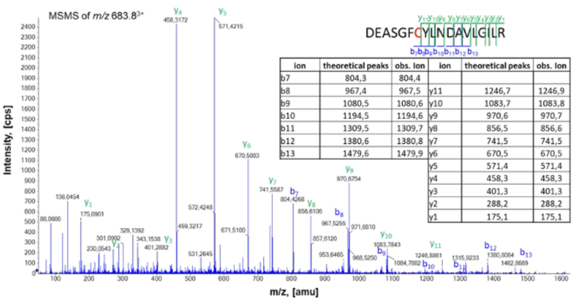

ion	theoretical peaks	obs. Ion	ion	theoretical peaks	obs. Ion
b7	804,3	804,4			
b8	967,4	967,5	y11	1246,7	1246,9
b9	1080,5	1080,6	y10	1083,7	1083,8
b10	1194,5	1194,6	y9	970,6	970,7
b11	1309,5	1309,7	y8	856,5	856,6
b12	1380,6	1380,8	y7	741,5	741,5
b13	1479,6	1479,9	y6	670,5	670,5
			y5	571,4	571,4
			y4	458,3	458,3
			y3	401,3	401,3
			y2	288,2	288,2
			y1	175,1	175,1

C6+

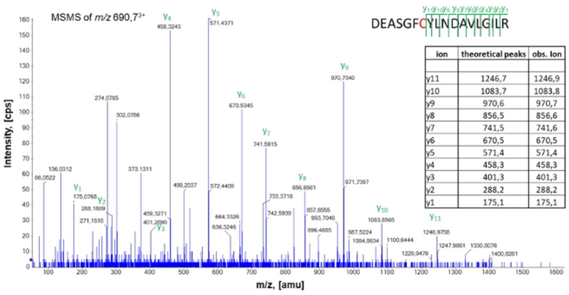

ion	theoretical peaks	obs. Ion
y11	1246,7	1246,9
y10	1083,7	1083,8
y9	970,6	970,7
y8	856,5	856,6
y7	741,5	741,6
y6	670,5	670,5
y5	571,4	571,4
y4	458,3	458,3
y3	401,3	401,3
y2	288,2	288,2
y1	175,1	175,1

D1+

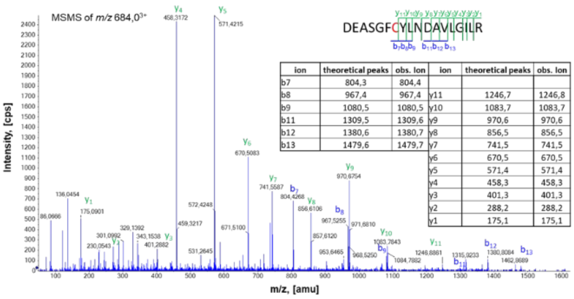

ion	theoretical peaks	obs. Ion	ion	theoretical peaks	obs. Ion
b7	804,3	804,4			
b8	967,4	967,4	y11	1246,7	1246,8
b9	1080,5	1080,5	y10	1083,7	1083,7
b11	1309,5	1309,6	y9	970,6	970,6
b12	1380,6	1380,7	y8	856,5	856,5
b13	1479,6	1479,7	y7	741,5	741,5
			y6	670,5	670,5
			y5	571,4	571,4
			y4	458,3	458,3
			y3	401,3	401,3
			y2	288,2	288,2
			y1	175,1	175,1

D2+

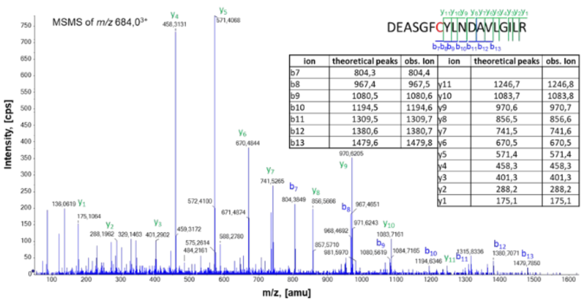

ion	theoretical peaks	obs. Ion	ion	theoretical peaks	obs. Ion
b7	804,3	804,4			
b8	967,4	967,5	y11	1246,7	1246,8
b9	1080,5	1080,6	y10	1083,7	1083,8
b10	1194,5	1194,6	y9	970,6	970,7
b11	1309,5	1309,7	y8	856,5	856,6
b12	1380,6	1380,7	y7	741,5	741,6
b13	1479,6	1479,8	y6	670,5	670,5
			y5	571,4	571,4
			y4	458,3	458,3
			y3	401,3	401,3
			y2	288,2	288,2
			y1	175,1	175,1

F2+

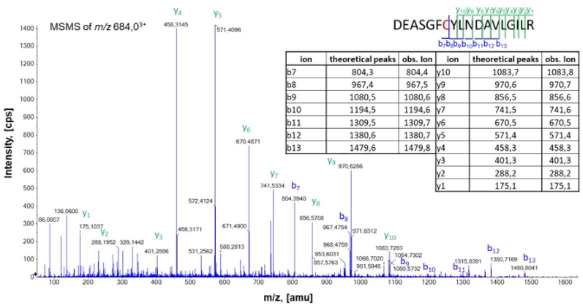

ion	theoretical peaks	obs. Ion	ion	theoretical peaks	obs. Ion
b7	804,3	804,4	y10	1083,7	1083,8
b8	967,4	967,5	y9	970,6	970,7
b9	1080,5	1080,6	y8	856,5	856,6
b10	1194,5	1194,6	y7	741,5	741,6
b11	1309,5	1309,7	y6	670,5	670,5
b12	1380,6	1380,7	y5	571,4	571,4
b13	1479,6	1479,8	y4	458,3	458,3
			y3	401,3	401,3
			y2	288,2	288,2
			y1	175,1	175,1

D3+

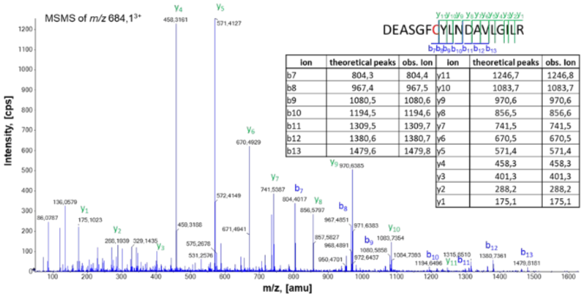

ion	theoretical peaks	obs. Ion	ion	theoretical peaks	obs. Ion
b7	804,3	804,4	y11	1246,7	1246,8
b8	967,4	967,5	y10	1083,7	1083,7
b9	1080,5	1080,6	y9	970,6	970,6
b10	1194,5	1194,6	y8	856,5	856,6
b11	1309,5	1309,7	y7	741,5	741,5
b12	1380,6	1380,7	y6	670,5	670,5
b13	1479,6	1479,8	y5	571,4	571,4
			y4	458,3	458,3
			y3	401,3	401,3
			y2	288,2	288,2
			y1	175,1	175,1

F3+

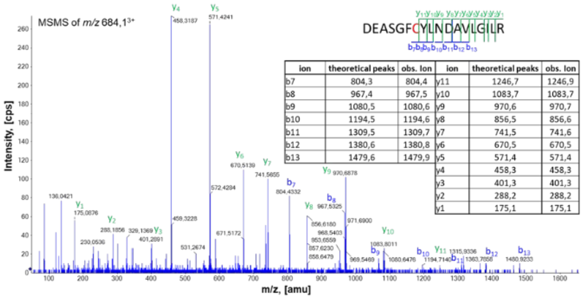

ion	theoretical peaks	obs. Ion	ion	theoretical peaks	obs. Ion
b7	804,3	804,4	y11	1246,7	1246,9
b8	967,4	967,5	y10	1083,7	1083,7
b9	1080,5	1080,6	y9	970,6	970,7
b10	1194,5	1194,6	y8	856,5	856,6
b11	1309,5	1309,7	y7	741,5	741,6
b12	1380,6	1380,8	y6	670,5	670,5
b13	1479,6	1479,9	y5	571,4	571,4
			y4	458,3	458,3
			y3	401,3	401,3
			y2	288,2	288,2
			y1	175,1	175,1

G6+

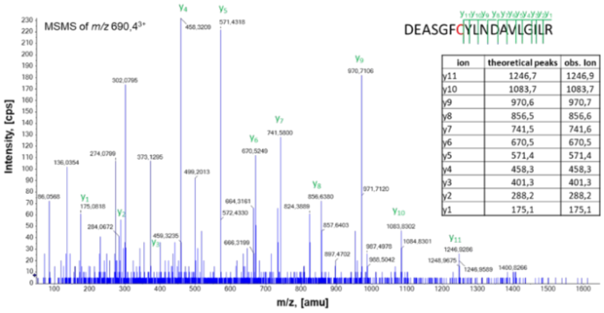

ion	theoretical peaks	obs. Ion
y11	1246,7	1246,9
y10	1083,7	1083,7
y9	970,6	970,7
y8	856,5	856,6
y7	741,5	741,6
y6	670,5	670,5
y5	571,4	571,4
y4	458,3	458,3
y3	401,3	401,3
y2	288,2	288,2
y1	175,1	175,1

B6+

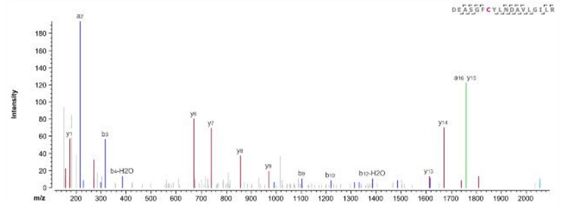

ion	theoretical peaks	obs. peaks
b2-H2O	227.0662479	227.0659
b3	316.1139263	316.118
b3-H2O	298.1033617	298.1024
b4-H2O	385.1353901	385.138
b8	990.4195782	990.387
b9	1103.503642	1103.48
b10	1217.54657	1217.507
b11	1332.573513	1332.52
b11-H2O	1314.562948	1314.528
b12-H2O	1385.600062	1385.597
b13-H2O	1484.668476	1484.651
b14	1615.763104	1615.729
y13	1613.90423	1613.846
y14	1670.925693	1670.899
y15-H2O	1739.947157	1739.902
y16-H2O	1810.984271	1810.949
y2-NH3	271.176467	271.1768
y6	670.4610217	670.4662
y7	741.4981355	741.5108
y8	856.5250785	856.5337
y9	970.568006	970.5461

3

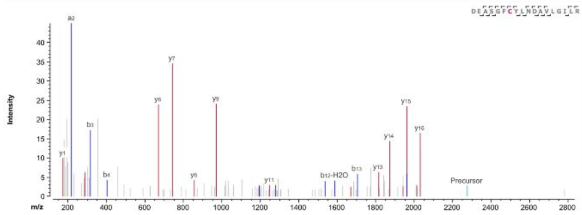

ion	theoretical peaks	obs. peaks
a2	217.0818979	217.0781
a9	1277.529572	1277.494
a16	1959.894562	1959.912
b3	316.1139263	316.1146
b4	403.1459548	403.142
b8	1192.440423	1192.475
b11	1534.594357	1534.588
b12-H2O	1587.620906	1587.624
b13	1704.699885	1704.693
y1	175.1189522	175.1166
y2	288.2030161	288.2023
y6	670.4610217	670.4671
y7	741.4981355	741.5035
y8	856.5250785	856.4903
y9	970.568006	970.6012
y11	1246.715398	1246.702
y12	1668.85666	1668.803
y13	1815.925074	1815.931
y14	1872.946538	1872.937
y15	1959.978566	1960.001
y15-H2O	1941.968001	1942.051
y16	2031.01568	2031.058
y16-H2O	2013.005115	2013.053

Supplementary Figure S8. The MSMS spectrum and annotation of **A3+**, **C3+**, **C6+**, **D1+**, **D2+**, **D3+** modified HDAC8 peptide [**241-250**]

A3+

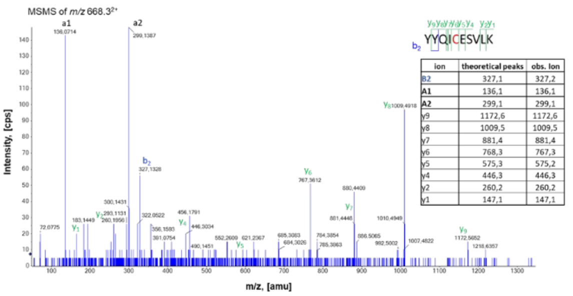

C3+

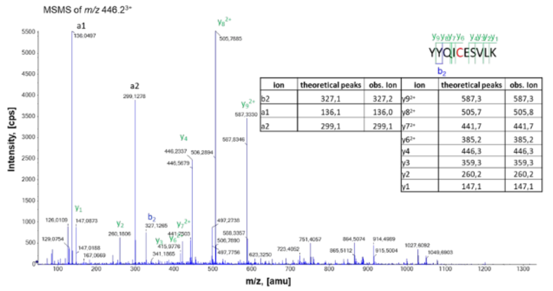

C6+

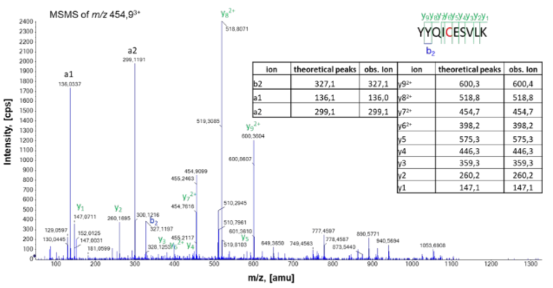

D1+

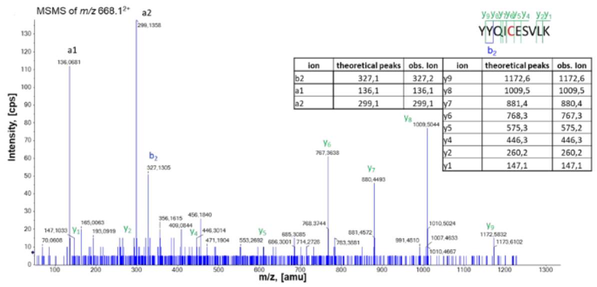

D2+

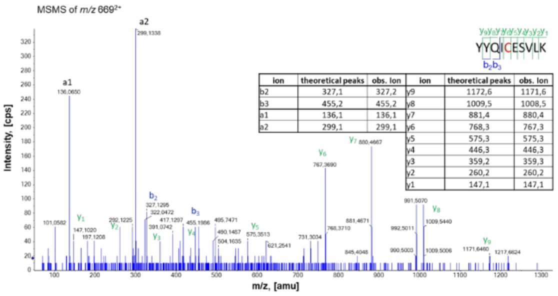

D3+

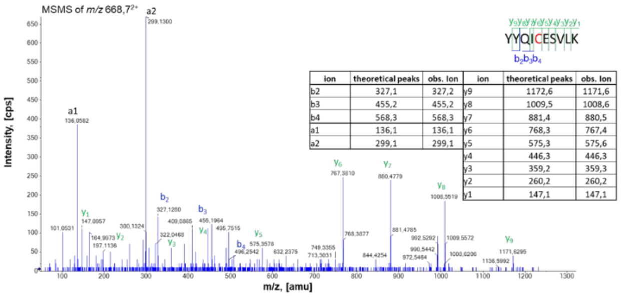

Supplementary Figure S9. The MSMS spectrum and annotation of **C3+**, **F2+**, **D3+**, **F3+**, **G6+** modified HDAC8 peptide [**260-287**]

C3+

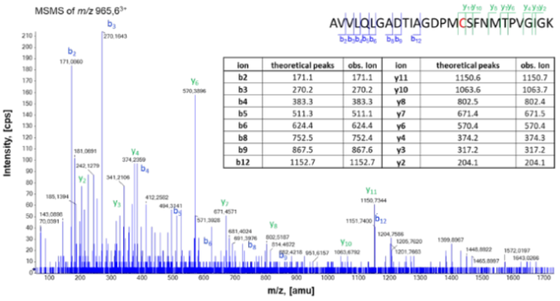

ion	theoretical peaks	obs. Ion	ion	theoretical peaks	obs. Ion
b2	171.1	171.1	y11	1150.6	1150.7
b3	270.2	270.2	y10	1063.6	1063.7
b4	383.3	383.3	y8	802.5	802.4
b5	511.3	511.1	y7	671.4	671.5
b6	624.4	624.4	y6	570.4	570.4
b8	752.5	752.4	y4	374.2	374.3
b9	867.5	867.6	y3	317.2	317.2
b12	1152.7	1152.7	y2	204.1	204.1

F2+

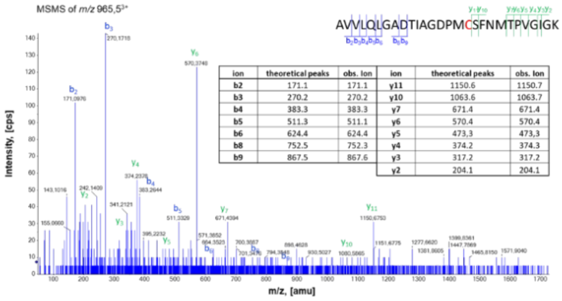

ion	theoretical peaks	obs. Ion	ion	theoretical peaks	obs. Ion
b2	171.1	171.1	y11	1150.6	1150.7
b3	270.2	270.2	y10	1063.6	1063.7
b4	383.3	383.3	y7	671.4	671.4
b5	511.3	511.1	y6	570.4	570.4
b6	624.4	624.4	y5	473,3	473,3
b8	752.5	752.3	y4	374.2	374.3
b9	867.5	867.6	y3	317.2	317.2
			y2	204.1	204.1

D3+

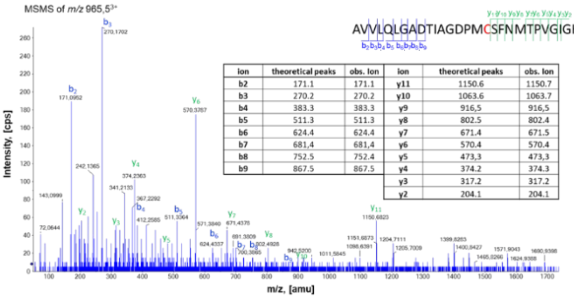

ion	theoretical peaks	obs. Ion	ion	theoretical peaks	obs. Ion
b2	171.1	171.1	y11	1150.6	1150.7
b3	270.2	270.2	y10	1063.6	1063.7
b4	383.3	383.3	y9	916,5	916,5
b5	511.3	511.3	y8	802.5	802.4
b6	624.4	624.4	y7	671.4	671.5
b7	681,4	681,4	y6	570.4	570.4
b8	752.5	752.4	y5	473,3	473,3
b9	867.5	867.5	y4	374.2	374.3
			y3	317.2	317.2
			y2	204.1	204.1

F3+

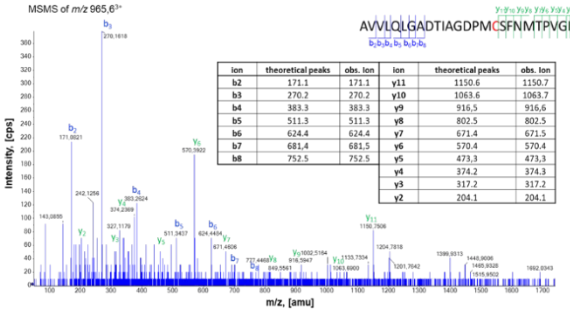

ion	theoretical peaks	obs. Ion	ion	theoretical peaks	obs. Ion
b2	171.1	171.1	y11	1150.6	1150.7
b3	270.2	270.2	y10	1063.6	1063.7
b4	383.3	383.3	y9	916,5	916,6
b5	511.3	511.3	y8	802.5	802.5
b6	624.4	624.4	y7	671.4	671.5
b7	681,4	681,5	y6	570.4	570.4
b8	752.5	752.5	y5	473,3	473,3
			y4	374.2	374.3
			y3	317.2	317.2
			y2	204.1	204.1

G6+

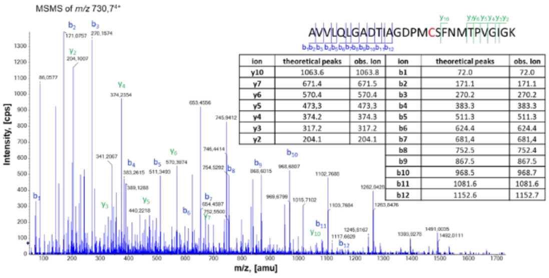

ion	theoretical peaks	obs. Ion	ion	theoretical peaks	obs. Ion
y10	1063.6	1063.8	b1	72.0	72.0
y7	671.4	671.5	b2	171.1	171.1
y6	570.4	570.4	b3	270.2	270.2
y5	473,3	473,3	b4	383.3	383.3
y4	374.2	374.3	b5	511.3	511.3
y3	317.2	317.2	b6	624.4	624.4
y2	204.1	204.1	b7	681,4	681,4
			b8	752.5	752.4
			b9	867.5	867.5
			b10	968.5	968.7
			b11	1081.6	1081.6
			b12	1152.6	1152.7

Supplementary Figure S10. The MSMS spectrum and annotation of **F3+** modified HDAC8 peptide [**327-357**]

F3+

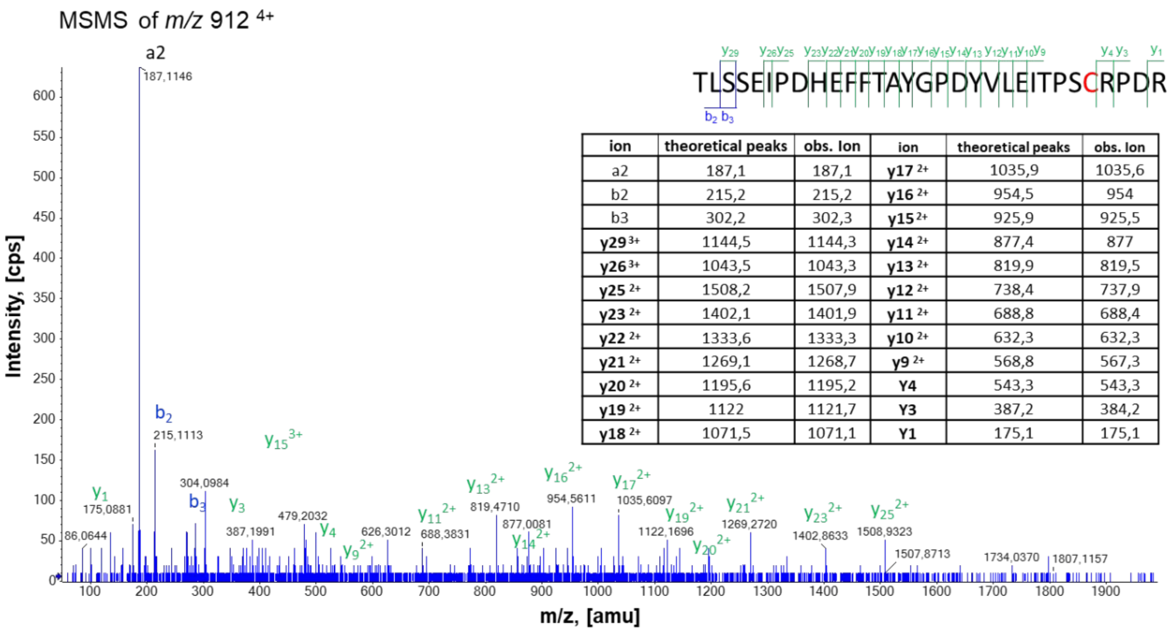

ion	theoretical peaks	obs. Ion	ion	theoretical peaks	obs. Ion
a2	187,1	187,1	**y17** 2+	1035,9	1035,6
b2	215,2	215,2	**y16** 2+	954,5	954
b3	302,2	302,3	**y15** 2+	925,9	925,5
**y29** 3+	1144,5	1144,3	**y14** 2+	877,4	877
**y26** 3+	1043,5	1043,3	**y13** 2+	819,9	819,5
**y25** 2+	1508,2	1507,9	**y12** 2+	738,4	737,9
**y23** 2+	1402,1	1401,9	**y11** 2+	688,8	688,4
**y22** 2+	1333,6	1333,3	**y10** 2+	632,3	632,3
**y21** 2+	1269,1	1268,7	**y9** 2+	568,8	567,3
**y20** 2+	1195,6	1195,2	**Y4**	543,3	543,3
**y19** 2+	1122	1121,7	**Y3**	387,2	384,2
**y18** 2+	1071,5	1071,1	**Y1**	175,1	175,1

Supplementary Figure S11. Results of the dilution experiments proving the irreversible covalent labeling for compounds **3** and **B6+**

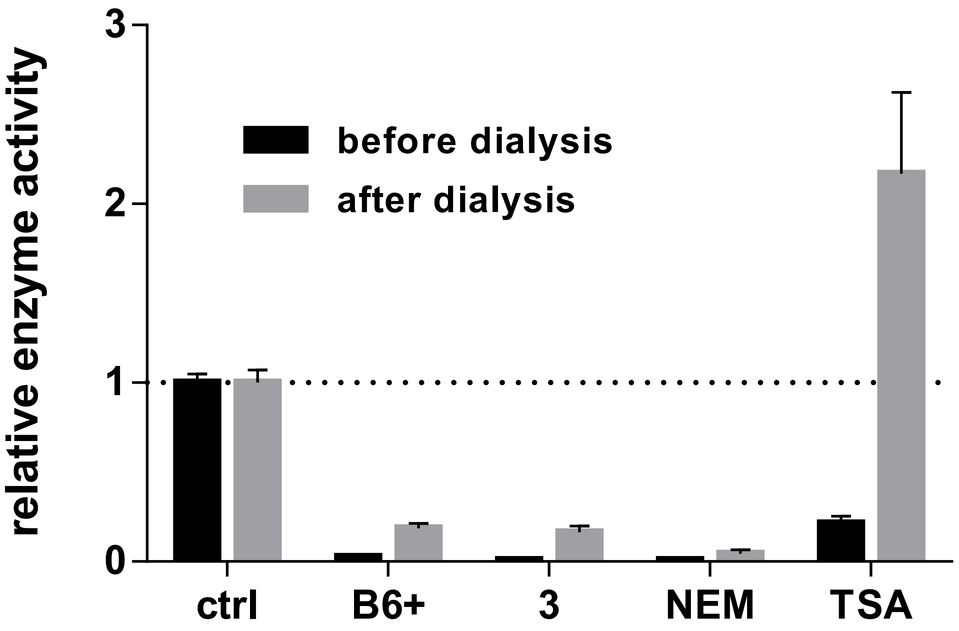

Reversibility of **B6+** and **3** on HDAC8 WT shown as relative enzyme activity before and after dialysis. Error bars represent mean +/- SD, n = 3

Supplementary Figure S12. MS Chromatogram for showing the labelling of GSH with **F4+**

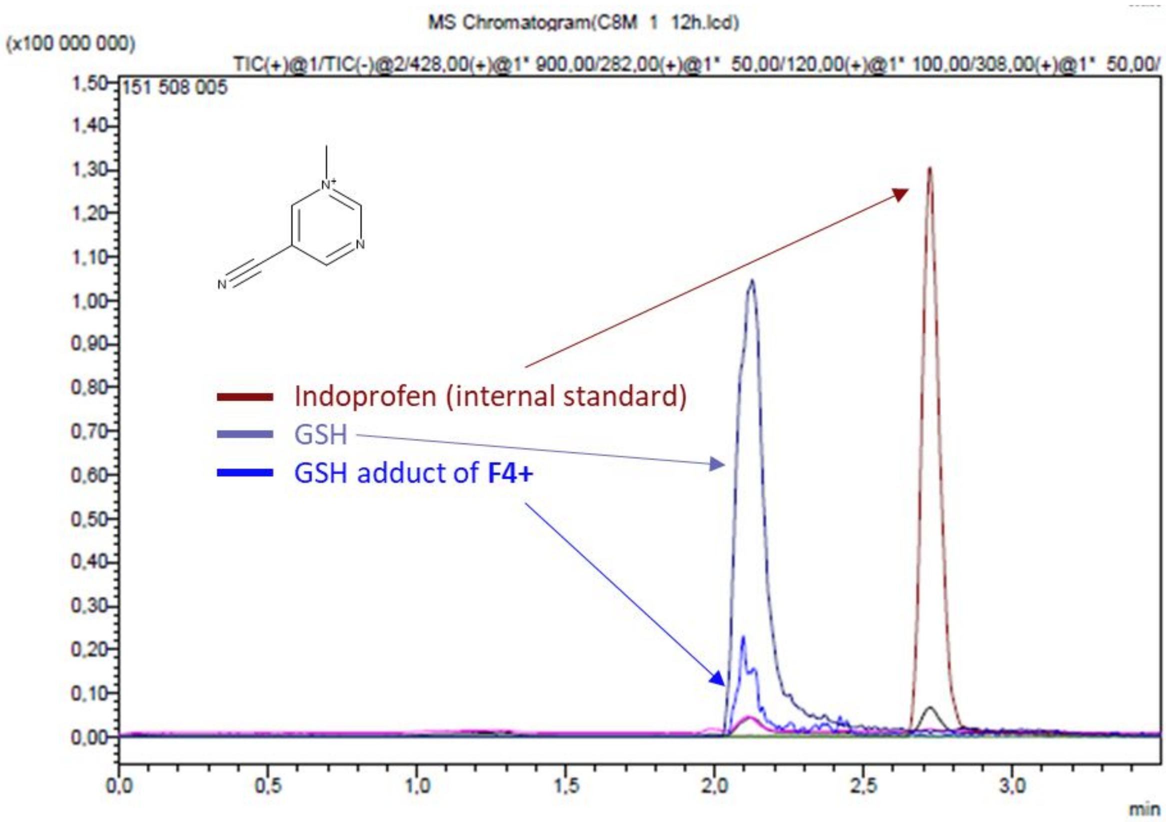

Supplementary Figure S13. Results of time dependent IC_{50} measurements and K_i, k_{inact}, k_{inact}/K_i calculations

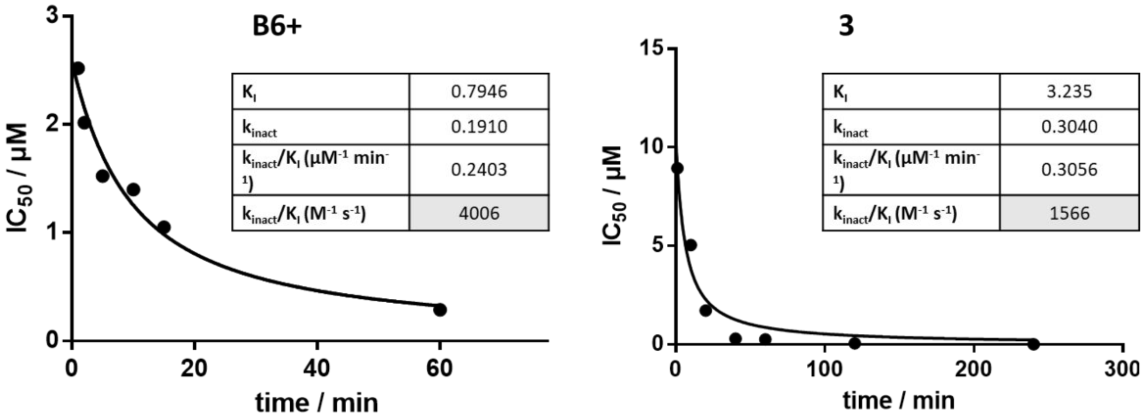

Supplementary Figure S14. Results of the western blot after 72 h treatment of OCI-AML3 cells

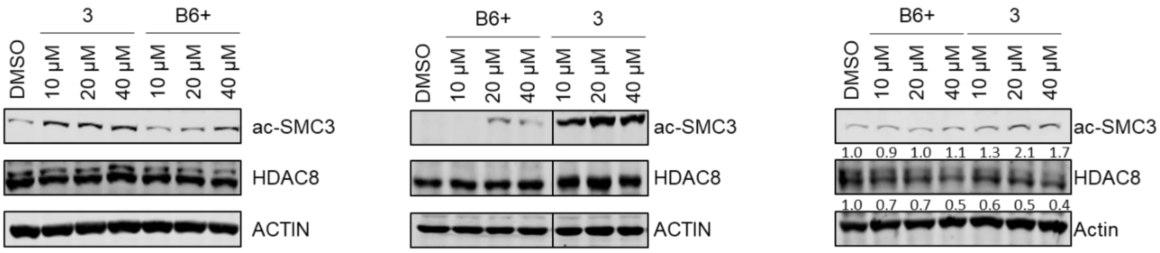

^{1}H, ^{13}C NMR spectra and HPLC-MS chromatogram and spectrum of **10**

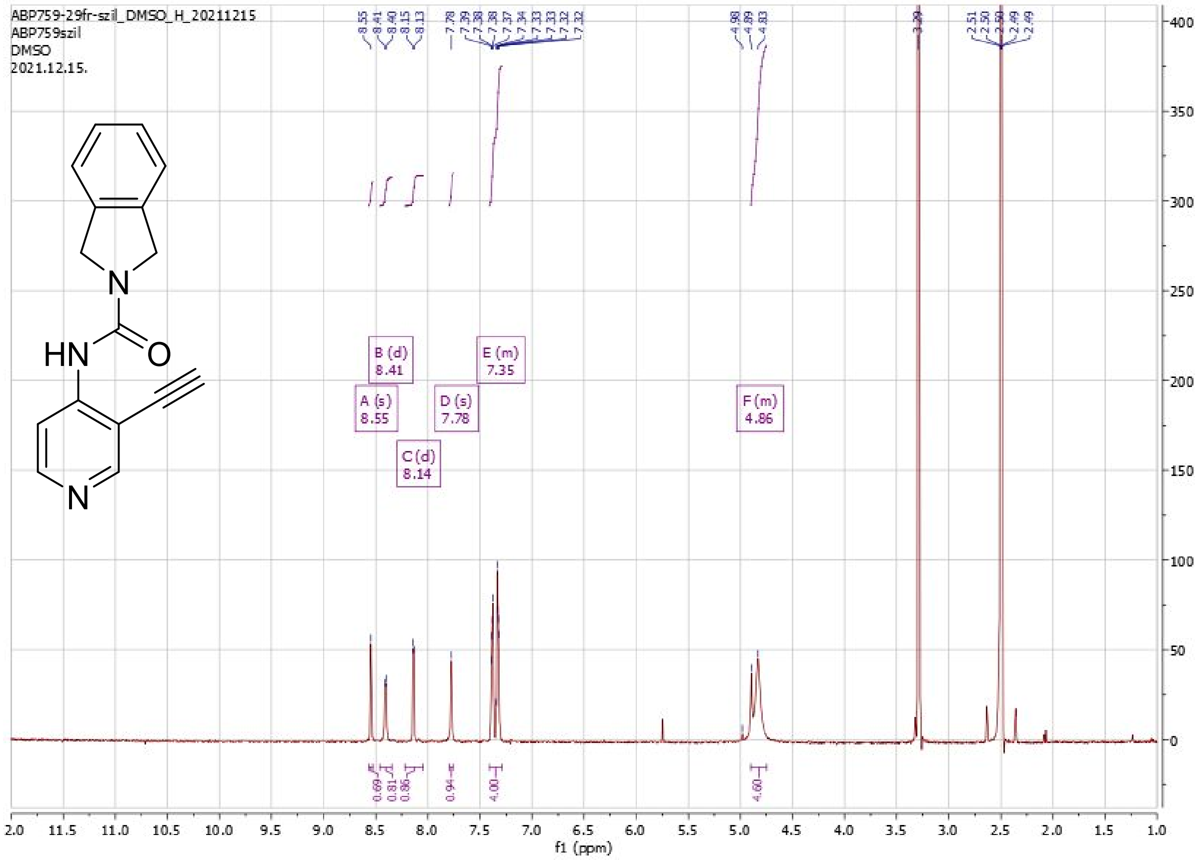

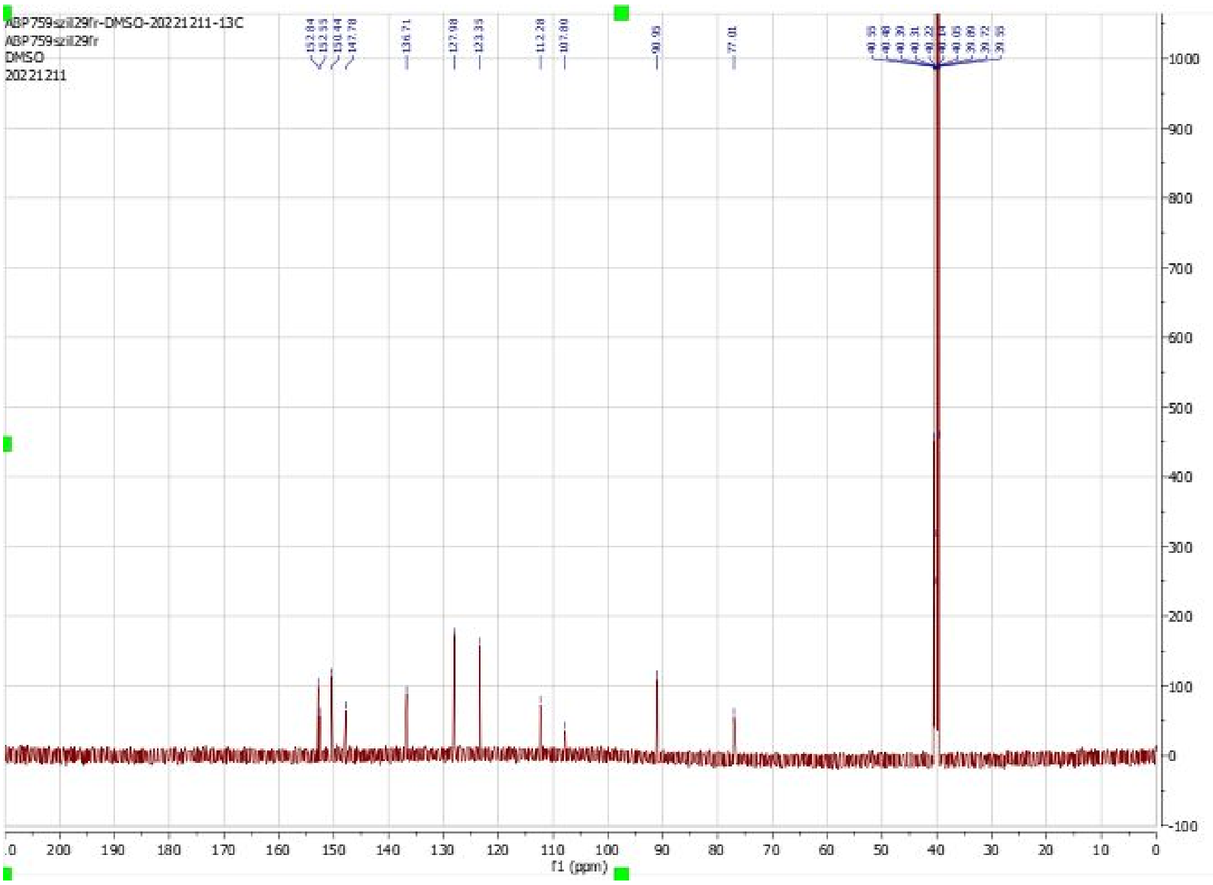

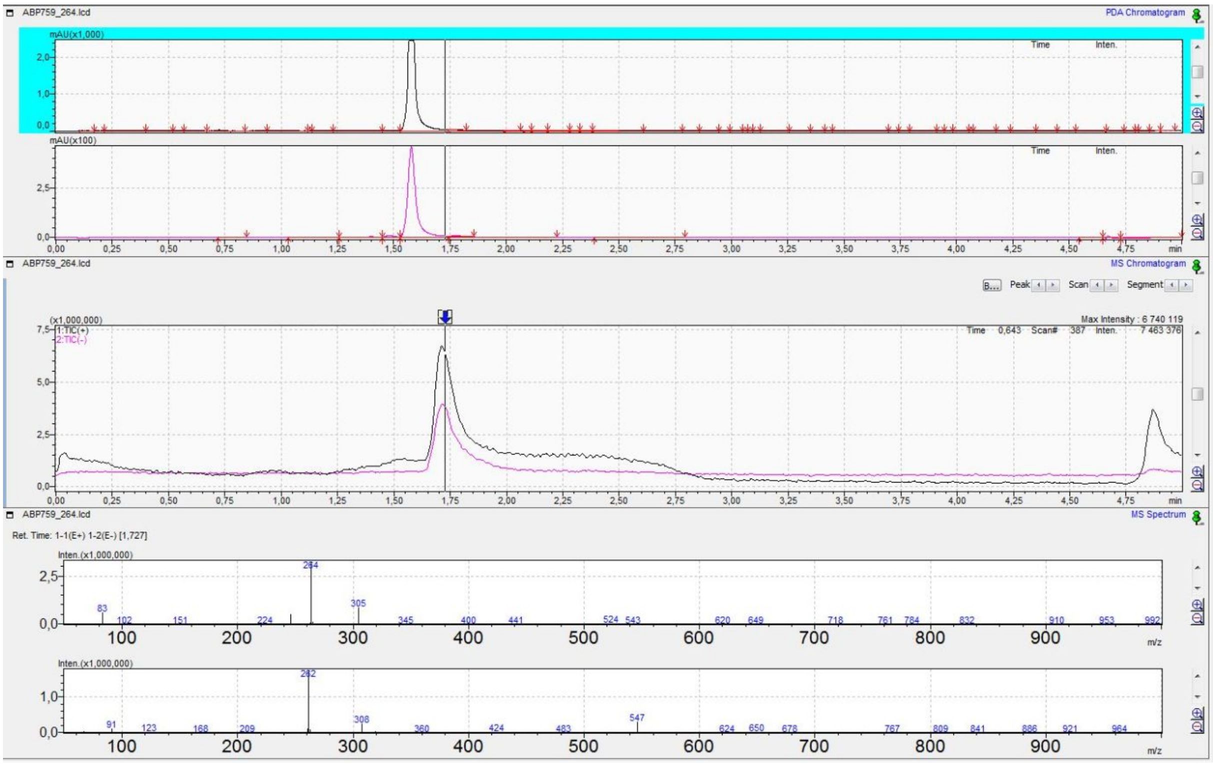

Peak#	Ret. Time	Height	Area	Area%
1	0.725	24741	37777	0.532
2	1.398	6182	14949	0.21
3	1.48	7303	13471	0.19
4	1.578	2451693	7026877	98.907
5	4.677	9501	11489	0.162
Total		2499419	7104563	100

Peak#	Ret. Time	Height	Area	Area%
1	0.731	1889	1712	0.14
2	1.481	7918	15921	1.302
3	1.579	442987	1189213	97.282
4	4.677	8151	15595	1.276
Total		460945	1222441	100

^{1}H, ^{13}C NMR spectra and HPLC-MS chromatogram and spectrum of **2**

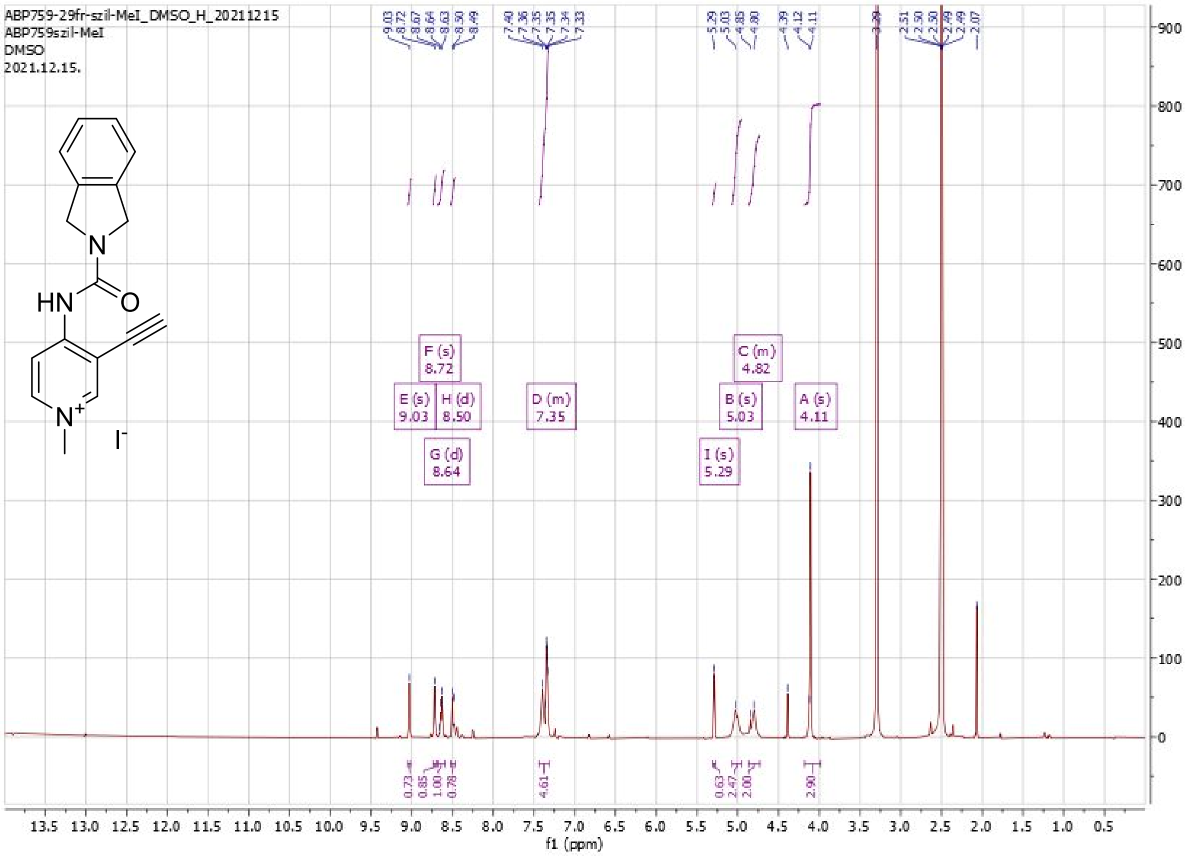

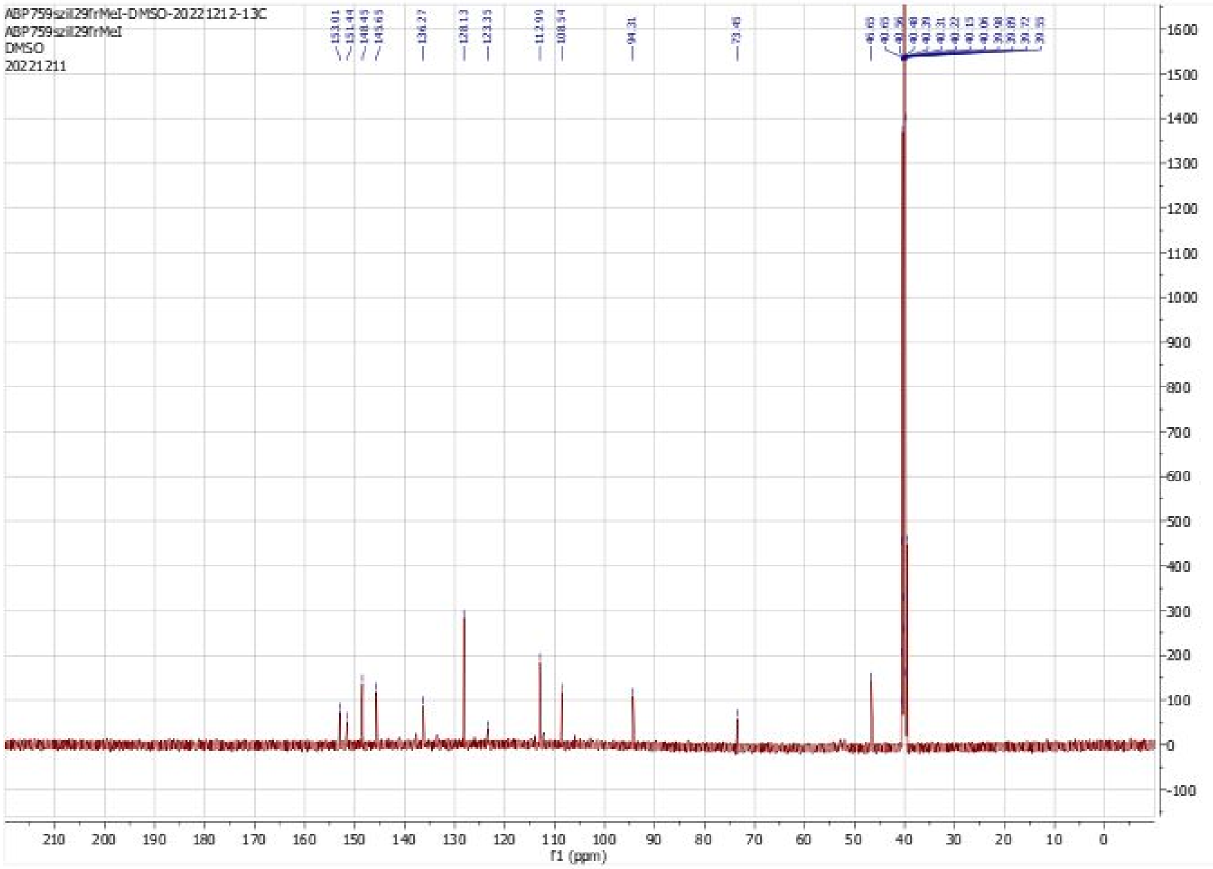

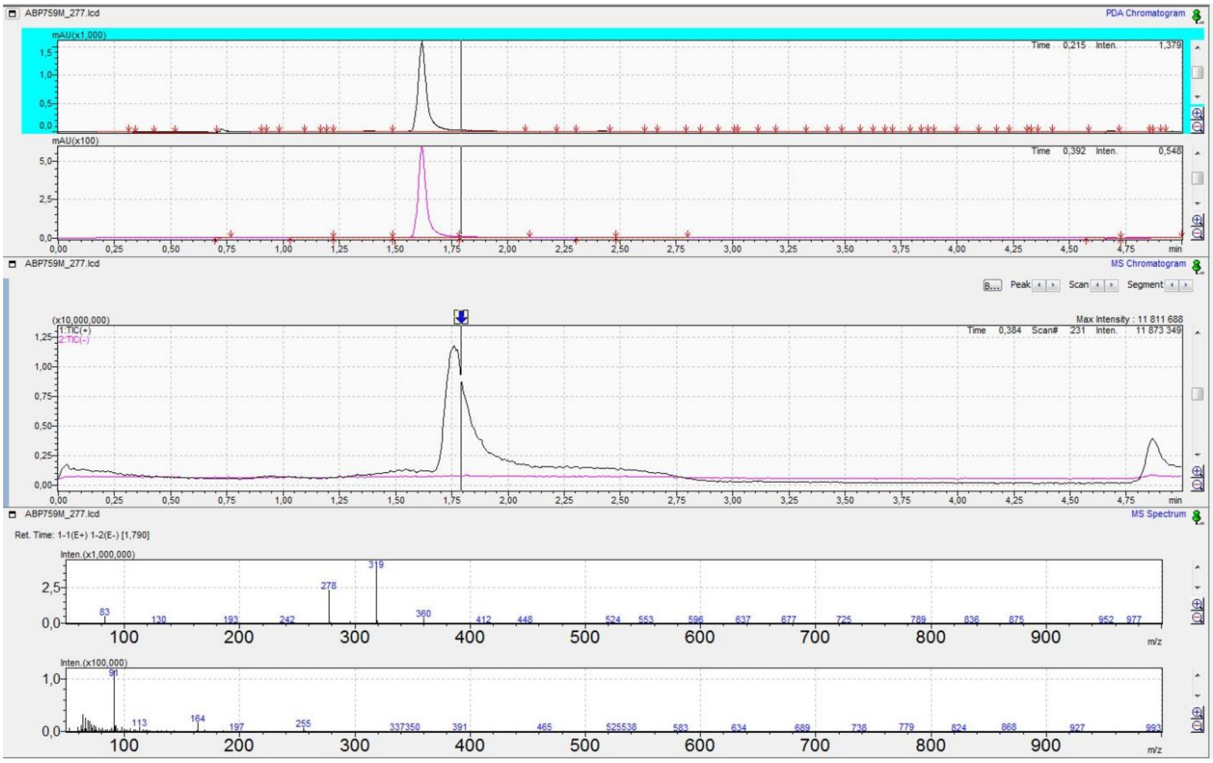

Peak#	Ret. Time	Height	Area	Area%
1	0.733	3107	2315	0.143
2	1.399	1313	5120	0.316
3	1.618	575510	1595585	98.365
4	2.536	1438	19094	1.177
Total		581368	1622114	100

Peak#	Ret. Time	Height	Area	Area%
1	0.729	48007	116486	2.516
2	1.397	8332	44423	0.959
3	1.618	1520747	4449011	96.093
4	2.428	4781	17338	0.374
5	4.692	7435	2642	0.057
Total		1589303	4629901	100

^{1}H, ^{13}C NMR spectra and HPLC-MS chromatogram and spectrum of **12**

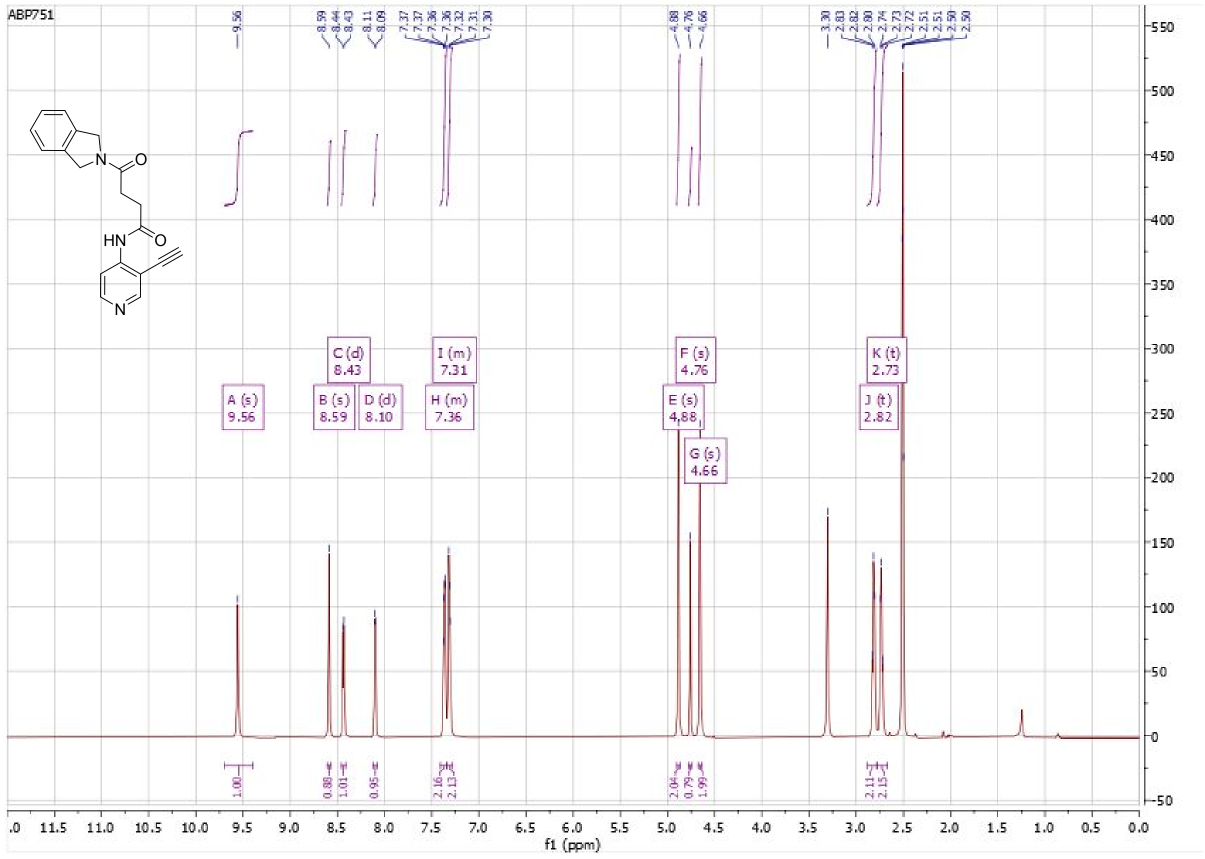

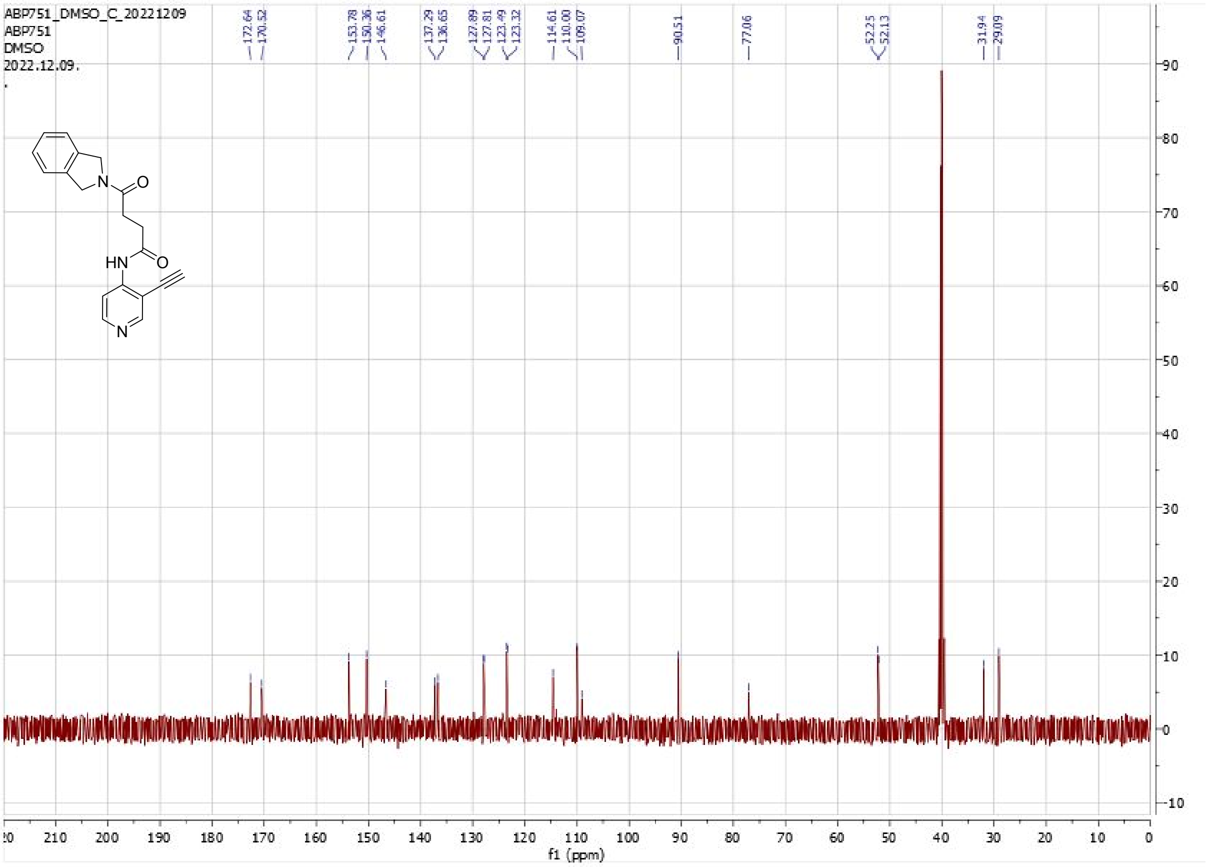

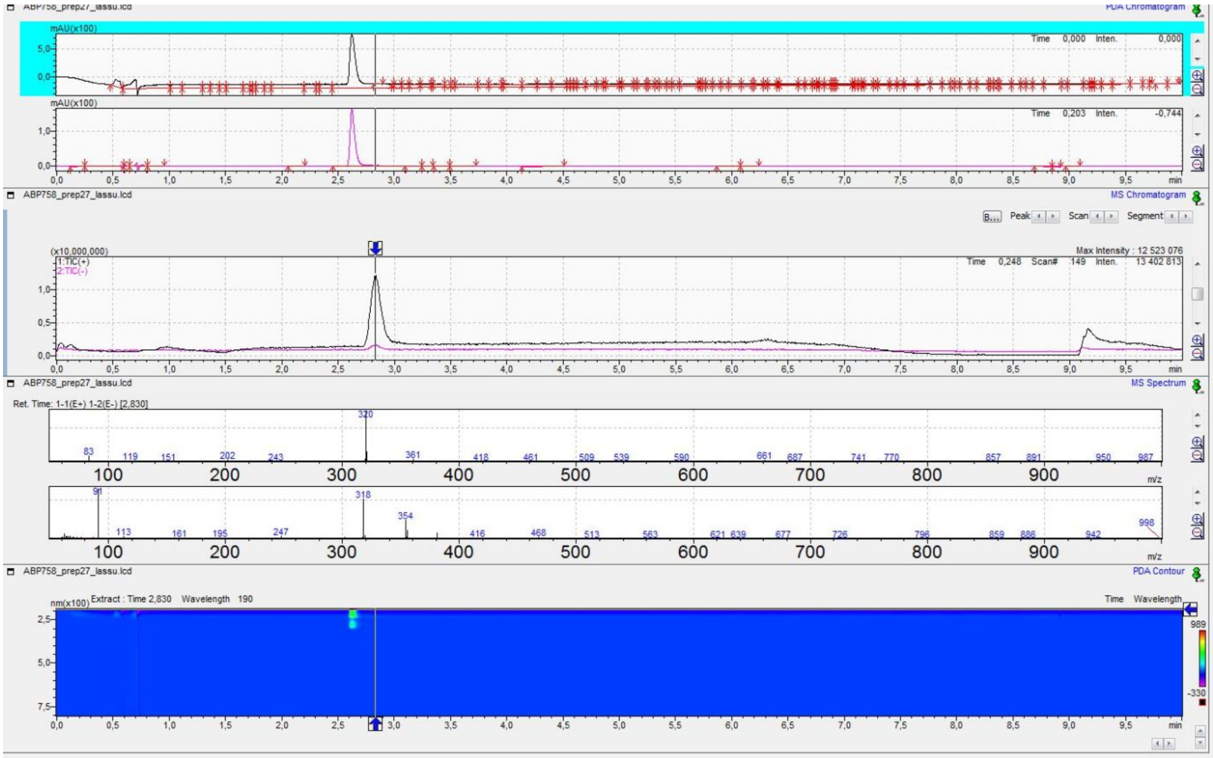

Peak#	Ret. Time	Height	Area	Area%	Peak#	Ret. Time	Height	Area	Area%
1	1.188	4855	19412	0.632	1	1.188	4855	19412	0.632
2	1.369	6076	32406	1.055	2	1.369	6076	32406	1.055
3	2.623	883032	3009149	97.981	3	2.623	883032	3009149	97.981
4	8.907	10740	10203	0.332	4	8.907	10740	10203	0.332
Total		904703	3071170	100	Total		904703	3071170	100

^{1}H, ^{13}C NMR spectra and HPLC-MS chromatogram and spectrum of **4**

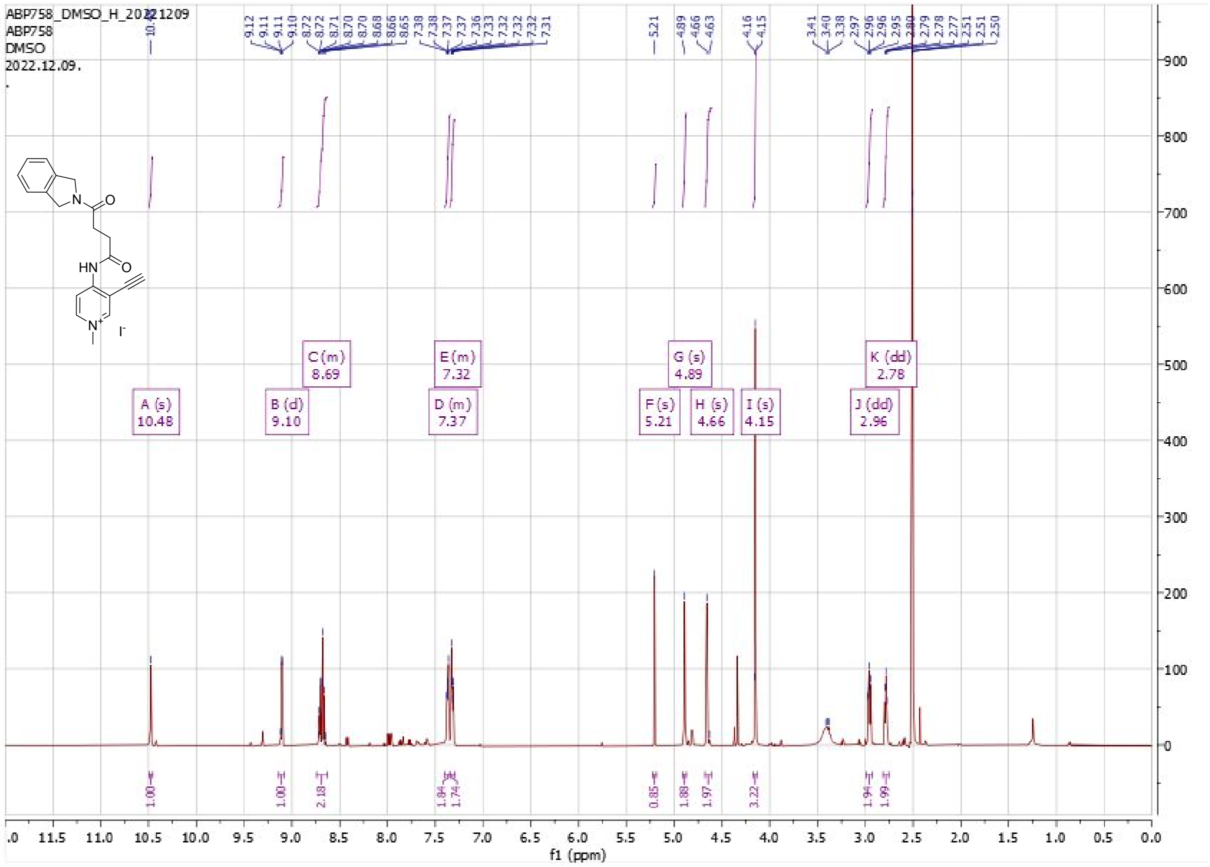

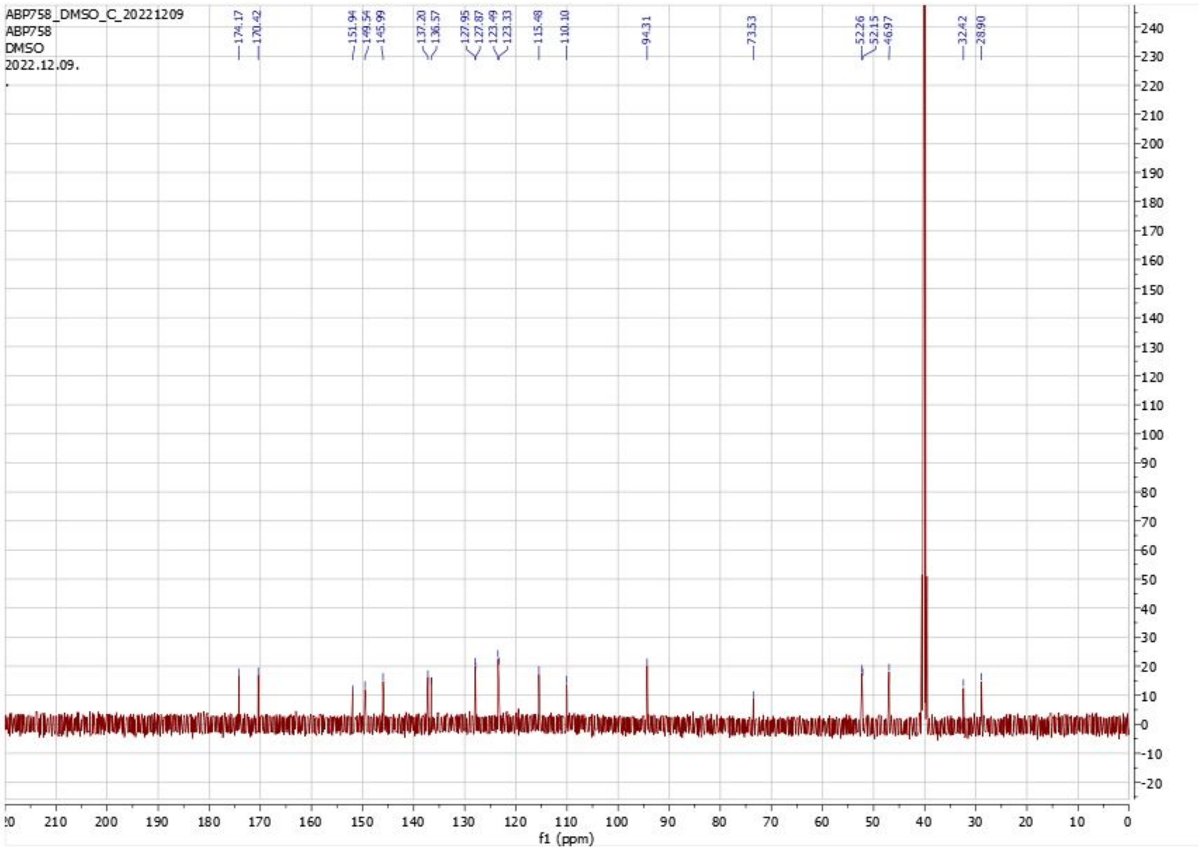

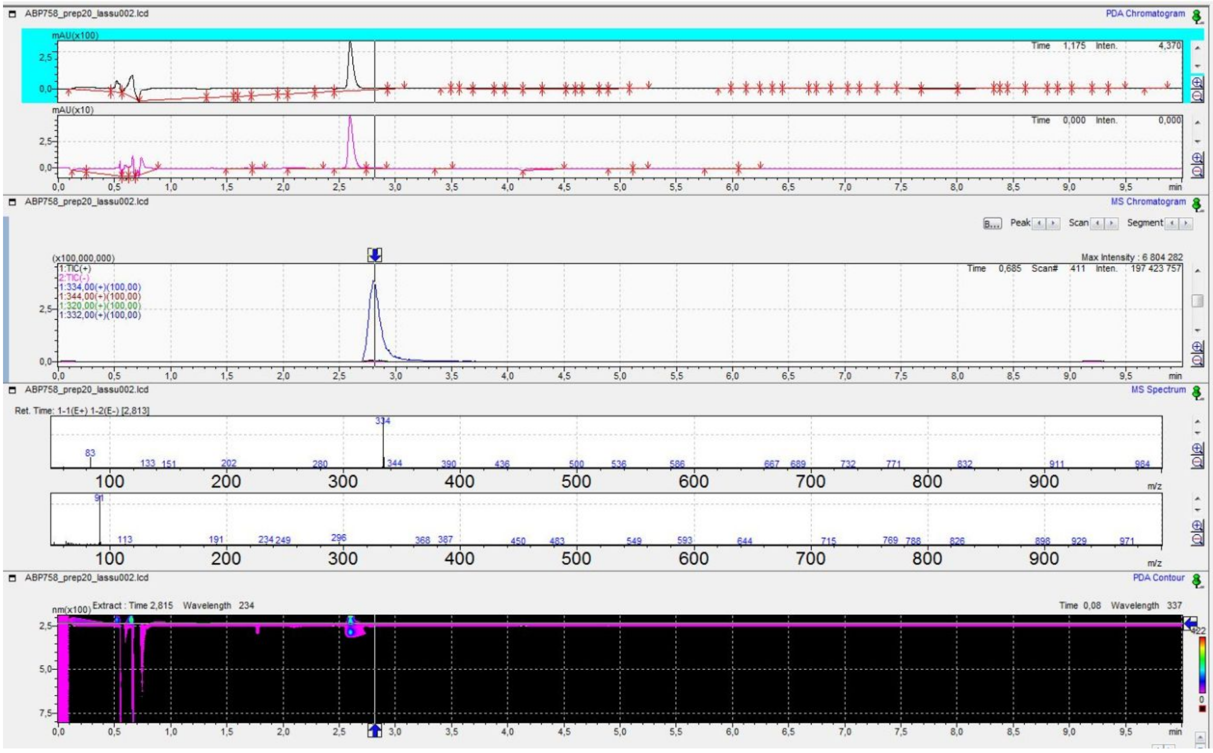

Peak#	Ret. Time	Height	Area	Area%	Peak#	Ret. Time	Height	Area	Area%
1	1.412	11222	55150	3.429	1	1.37	165	230	0.149
2	1.851	277	-634	-0.039	2	1.773	546	1103	0.713
3	2.108	4136	13261	0.825	3	2.099	429	1465	0.948
4	2.598	471237	1539452	95.727	4	2.598	48676	151770	98.19
5	7.587	1097	932	0.058	Total		49816	154568	100
Total		487970	1608161	100					

^{1}H, ^{13}C NMR spectra and HPLC-MS chromatogram and spectrum of **11**

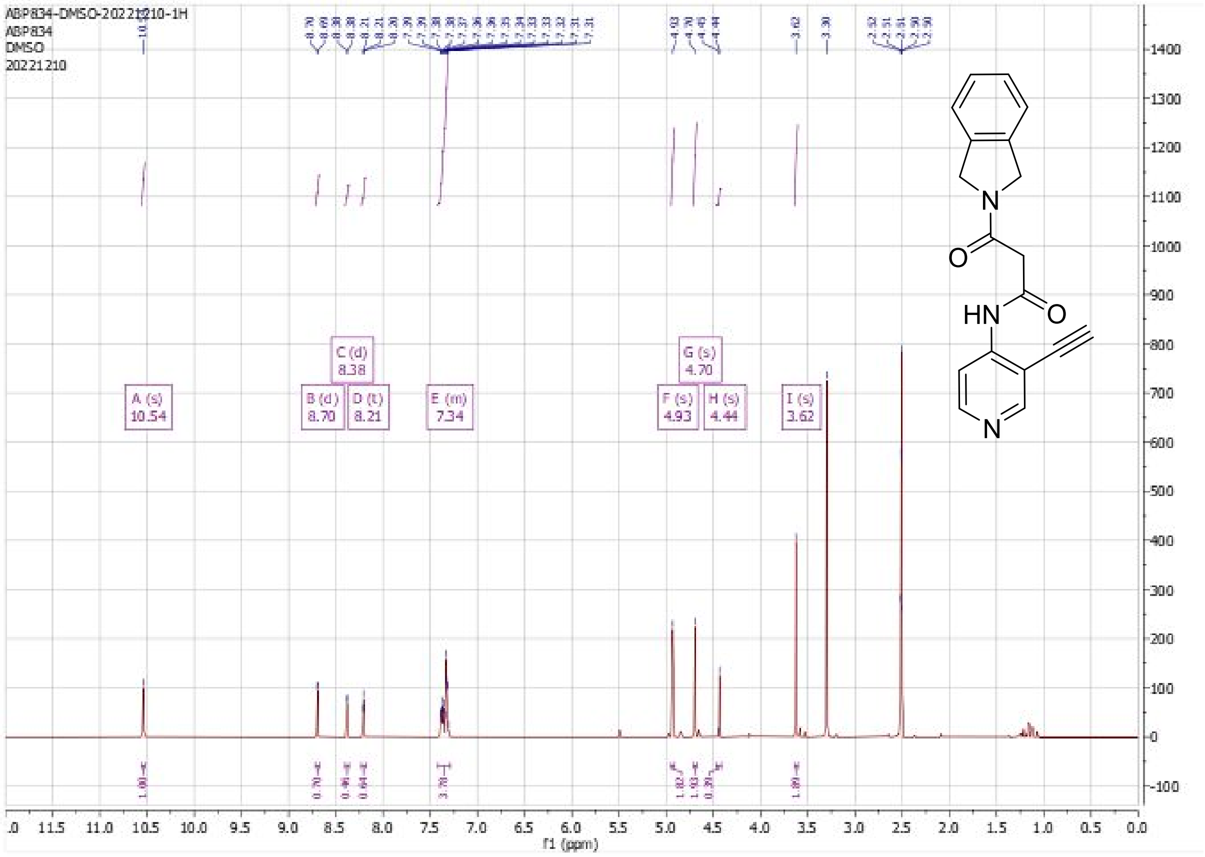

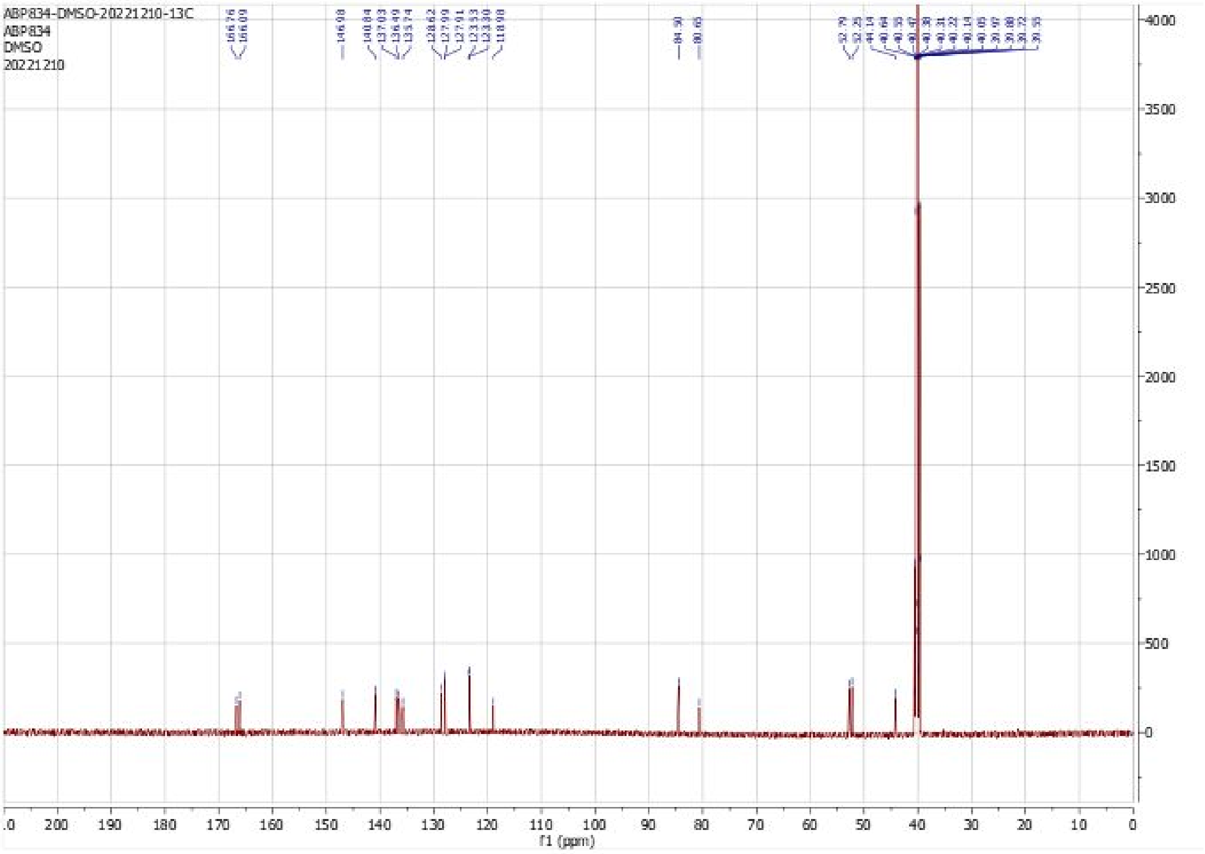

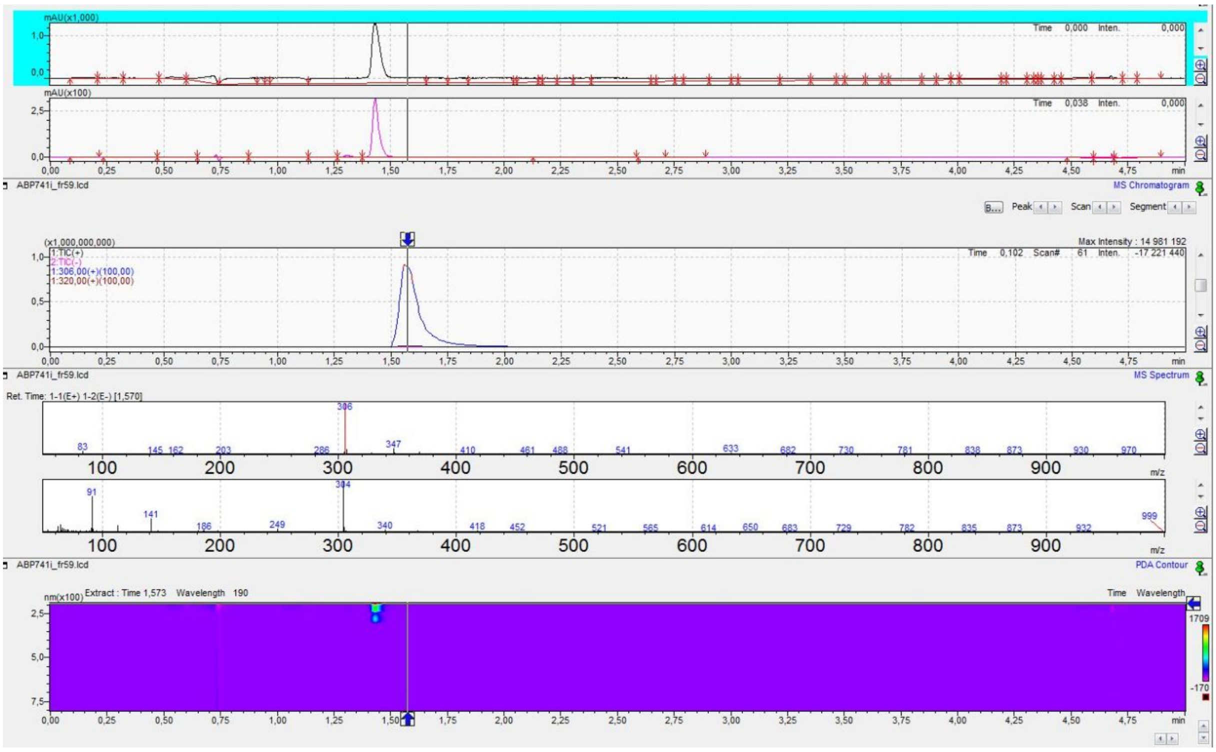

Peak#	Ret. Time	Height	Area	Area%	Peak#	Ret. Time	Height	Area	Area%
1	0.714	22833	46626	1.416	1	0.777	3516	7793	1.216
2	1.082	15914	48991	1.488	2	1.309	8676	17482	2.728
3	1.432	1266827	3197446	97.096	3	1.432	294473	615542	96.056
Total		1305574	3293063	100	Total		306664	640818	100

^{1}H, ^{13}C NMR spectra and HPLC-MS chromatogram and spectrum of **3**

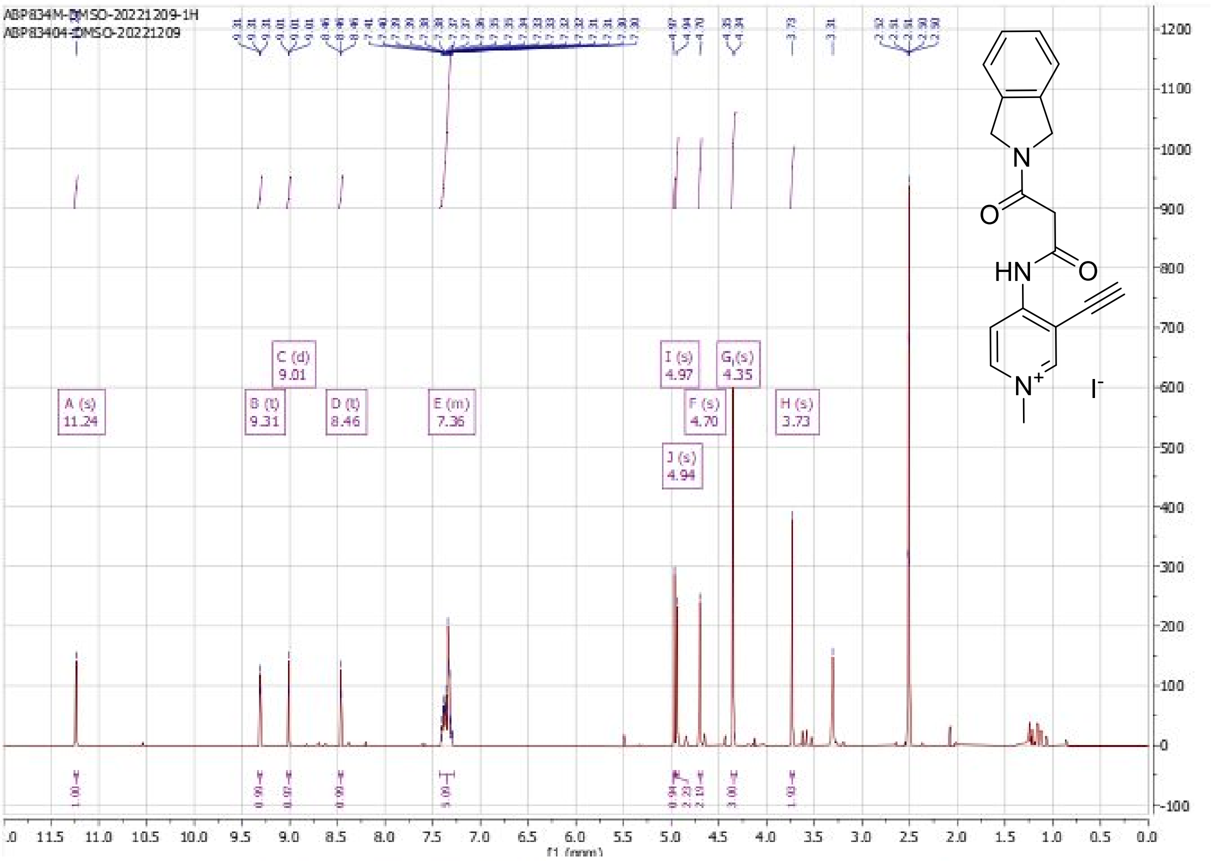

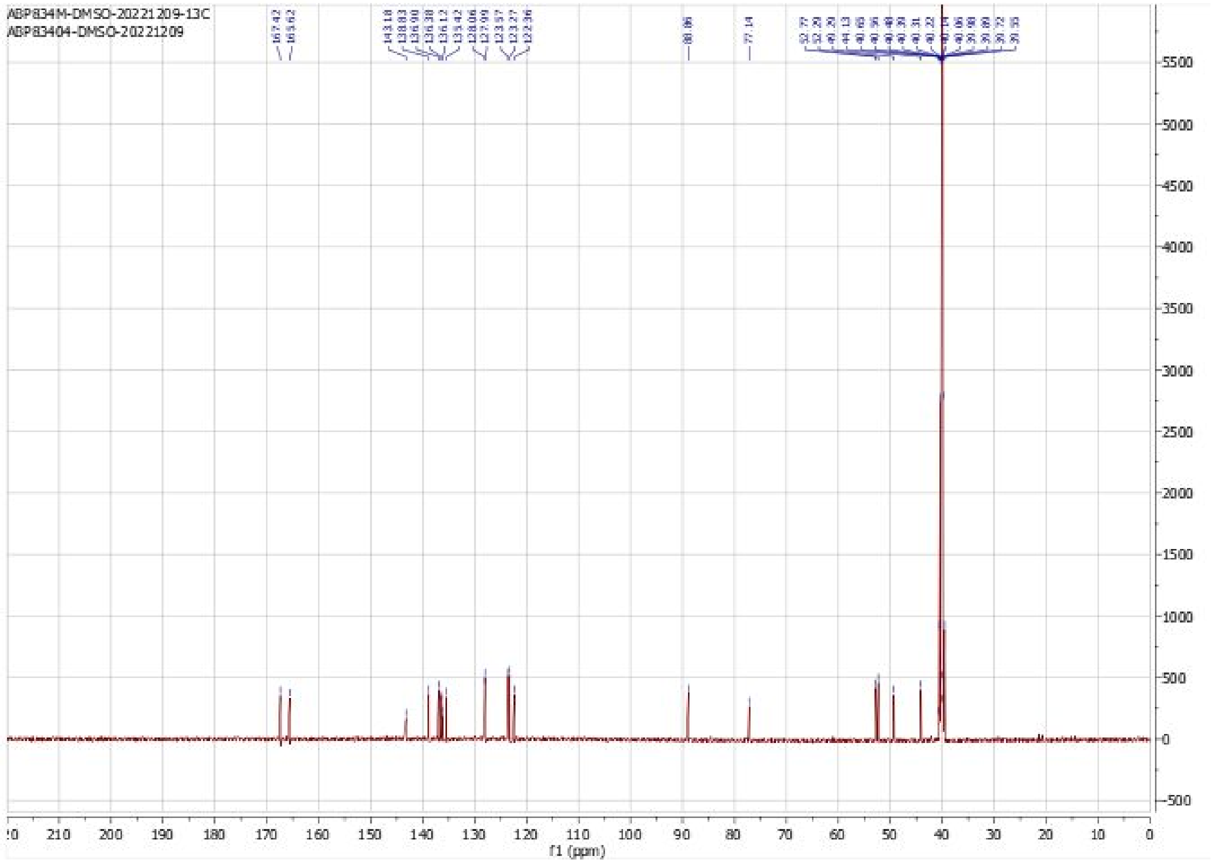

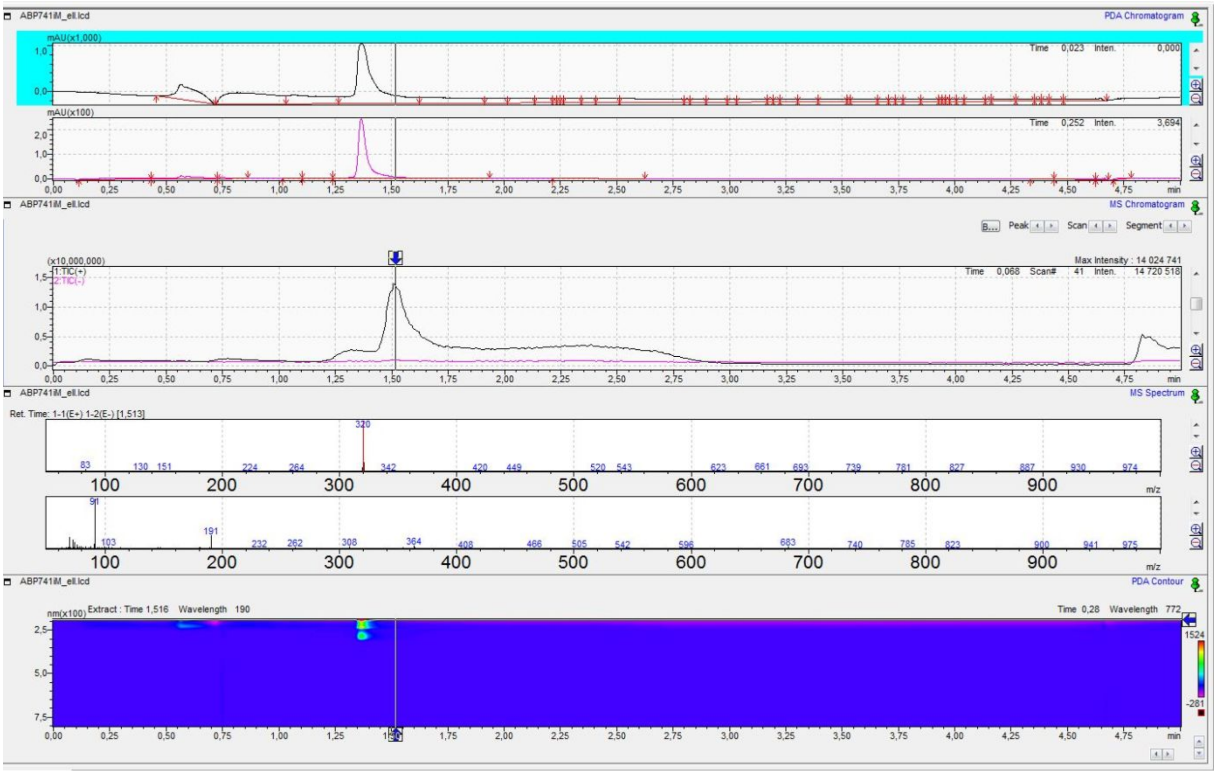

Peak#	Ret. Time	Height	Area	Area%	Peak#	Ret. Time	Height	Area	Area%
1	0.566	92037	113344	2.367	1	0.597	648	751	0.116
2	1.065	23731	105671	2.207	2	0.75	5234	11604	1.796
3	1.368	1130888	4558667	95.201	3	1.367	233787	633822	98.088
4	1.666	5801	10776	0.225	Total		239670	646177	100
Total		1252457	4788458	100					

Supplementary Tables S1, S2, S3

Supplementary tables.7z
